# Redesigning Medicaid frailty algorithms: improved identification of medically frail adults under community engagement

**DOI:** 10.1093/haschl/qxag108

**Published:** 2026-05-08

**Authors:** Sanjay Basu, Seth A Berkowitz

**Affiliations:** Department of Medicine, University of California SanFrancisco, San Francisco, CA 94158, United States; Waymark, SanFrancisco, CA 94115, United States; Division of General Medicine and Clinical Epidemiology, Department of Medicine, University of North Carolina at Chapel Hill, Chapel Hill, NC 27599, United States

**Keywords:** Medicaid, frailty, community engagement requirements, work requirements, Section 1115 waivers, algorithmic fairness, health equity, claims-based algorithms, microsimulation, ICD-10, health information exchange, ex parte determination, functional disability, coverage loss, One Big Beautiful Bill Act

## Abstract

Between 2018 and 2024, 14 US states sought or obtained Section 1115 waivers to condition Medicaid expansion coverage on community engagement requirements, with medically frail exemptions determined from claims-based administrative data; the One Big Beautiful Bill Act of 2025 (Public Law 119-21) codified these requirements nationally, with state implementation due by January 2027. Using American Community Survey Public Use Microdata Sample data from 75 043 Medicaid-enrolled adults aged 19-64 across 17 states, we simulated frailty identification under each state's existing algorithm via a 3-channel Monte Carlo microsimulation incorporating algorithm design, claims visibility, and documentation burden. Existing algorithms identified a mean of 31.4% of adults with functional disability as medically frail (range: 14.3% [Florida, Arizona] to 45.4% [New York]). An evidence-based redesigned algorithm incorporating expanded diagnostic criteria, health information exchange integration, ex parte determination, and elimination of physician certification requirements increased mean identification to 45.6% (+14.3% points), with gains across all 17 states. In multi-dimensional equity evaluation, the redesigned algorithm narrowed the American Indian/Alaska Native–White sensitivity gap by 46% (from 11.6% to 6.3% points) and the Black-White gap by 10% (from 12.8% to 11.5% points); within-race rural-urban sensitivity differences of 3%-5% points persisted under both algorithms. Adoption of the redesigned algorithm would identify an estimated 3.8 million additional medically frail adults and avert approximately 253 000 coverage losses under full implementation. These findings support minimum algorithmic design standards for state frailty determination systems.

## Introduction

Between 2018 and 2024, 14 US states sought or obtained Section 1115 waivers from the Centers for Medicare and Medicaid Services (CMS) to condition Medicaid coverage for some individuals on community engagement requirements—typically 80 hours per month of work, job training, education, or qualifying volunteer activity.^1,2^ Each waiver required identification and exemption of individuals classified as medically frail. The One Big Beautiful Bill Act of 2025 (Public Law 119-21) codified community engagement requirements nationally, mandating that all states implement them with medically frail exemptions by January 1, 2027.^3,4^ Under CMS guidance and individual waiver terms, states use claims-based administrative data—primarily Medicaid Management Information System (MMIS) files—to identify qualifying individuals from ICD-10 diagnosis code density, service counts, or validated claims-based frailty indices.^5^

State frailty algorithms vary substantially in design. Florida recognizes 5 ICD-10 diagnostic families and requires both physician certification and 2 or more ADL impairments; California recognizes 13 families and uses automated claims-based identification with health information exchange (HIE) integration.^5^ These differences reflect the fact that most state frailty algorithms were adapted from tools developed for mortality prediction or spending risk adjustment, not for identifying functional limitation among working-age adults.^6^ Algorithms designed to predict healthcare costs systematically under-predict the needs of populations with lower baseline healthcare utilization, as Obermeyer and colleagues demonstrated in a widely-cited analysis of a commercial risk stratification algorithm used on approximately 200 million patients per year.^7^ We cite this example because it establishes the *mechanism* by which any claims-based algorithm calibrated on utilization data systematically under-identifies individuals with lower baseline utilization—a structural property directly applicable to Medicaid frailty tools when applied to working-age populations who face structural barriers to care access, generating fewer qualifying codes regardless of true clinical need.

This mechanism—that claims-based tools miss individuals whose conditions are real but under-documented in administrative data—suggests that existing state frailty algorithms may under-identify medically frail adults overall. Under-documentation in claims data has well-established racial differentials: Black patients with equivalent disease burden use fewer ambulatory care services, receive fewer specialist referrals, and have more fragmented records distributed across safety-net providers.^7–9^ If algorithm redesign addresses the root causes of under-documentation, it could simultaneously improve the overall accuracy of frailty identification and narrow disparities across populations.

The first state-level implementation in Arkansas resulted in 18 164 individuals losing coverage by June 2019, with sustained coverage and e mployment effects at two-year follow-up.^10,11^ Judicial review of these waivers in Stewart v. Azar blocked the Kentucky implementation.^12^ The Kaiser Family Foundation (KFF) and the Medicaid and CHIP Payment and Access Commission (MACPAC) have documented wide variation in overall exemption rates across states, from below 8% to above 25%.^1,2^ No prior study has evaluated whether existing state frailty algorithms adequately identify medically frail adults or specified an evidence-based redesign.

This study (1) estimates the sensitivity of existing state frailty algorithms for identifying adults with functional disability, (2) specifies an evidence-based redesigned algorithm incorporating best practices from existing state implementations, (3) compares existing and redesigned algorithms in a head-to-head microsimulation, (4) evaluates whether sensitivity improvements also narrow disparities across race/ethnicity and rurality, and (5) projects the population-level coverage impact of algorithmic redesign.

## Methods

### Study design and data sources

This is a microsimulation analysis of 17 US states: 14 with active, pending, blocked, or terminated Medicaid community engagement requirement programs as of early 2024, and 3 comparator states (California, New York, North Carolina) with Medicaid expansion but no community engagement requirements, included to represent the range of existing frailty identification infrastructure. This study followed the STROBE statement and the RECORD extension for studies using routinely-collected health data.^13,14^

Individual-level data on functional disability among Medicaid enrollees were obtained from the American Community Survey (ACS) Public Use Microdata Sample (PUMS) 2022 1-year file. We identified 75 043 adults aged 19-64 enrolled in Medicaid with data on 6 disability domains (hearing, vision, cognitive, mobility, self-care, and independent living), race/ethnicity (non-Hispanic White, non-Hispanic Black, Hispanic, American Indian/Alaska Native [AIAN], Asian, and other), and geographic Public Use Microdata Area (PUMA) codes (eAppendix A1) (To access the appendix, click on the Details tab of the article online). Metropolitan status (metro vs nonmetro) was assigned using the IPUMS MSA-PUMA crosswalk linking 2020 PUMAs to 2023 metropolitan statistical areas; 86% of the sample was classified as metro and 14% as non-metro. State frailty policy characteristics—including ICD-10 diagnostic families recognized, ADL threshold, physician certification requirement, ex parte determination, HIE integration, and claims lag—were compiled from waiver documents and coded into a structured database of 17 state-specific definitions (eAppendix A2) (To access the appendix, click on the Details tab of the article online). Provider intensity data were derived from the HHS Medicaid Provider Spending Dataset (T1019 personal care services HCPCS Level II billing records); these data were used exclusively as an ecological correlate for geographic provider-density analyses and did not enter the main frailty microsimulation algorithm (eAppendix A3). The CDC Behavioral Risk Factor Surveillance System (BRFSS) Disability and Health Data System provided ecological disability prevalence estimates by state and race (eAppendix A4). All frailty identification rates reported in this study are derived from the microsimulation rather than directly observed in claims data. The status quo base case is constructed by applying each state's frailty determination algorithm to ACS PUMS individuals through the three-channel microsimulation parameterized from published evidence on race-differential healthcare utilization; simulated status quo identification rates are validated against state-reported exemption rates from KFF administrative data (eAppendices A5 and A6).

### Three-channel microsimulation

We modeled frailty identification as a 3-stage process reflecting real-world determination pathways:

#### Channel A (algorithm design)

For each individual, clinical eligibility was determined by whether their ACS-reported disability mapped to conditions recognized by the state's ICD-10 diagnostic list and whether their functional limitation met the state's ADL threshold. ACS disability domains were mapped to ICD-10 diagnostic families using a structured crosswalk (eAppendix A1): ambulatory difficulty (DPHY) maps to musculoskeletal [M00–M99] and nervous system [G10–G99] conditions; cognitive difficulty (DREM) maps to behavioral health [F20–F48] and neurological conditions; self-care difficulty (DDRS) maps to neurological and musculoskeletal conditions; independent living difficulty (DOUT) maps to any of the above plus social determinant codes; hearing and vision difficulty map to sensory [H60–H95] and ophthalmological [H00–H59] conditions, respectively. An individual is deemed clinically eligible under Channel A if their ACS disability domain(s) map to at least one ICD-10 family recognized by the state's algorithm and their reported functional limitations meet the state's ADL threshold. Because the ACS does not capture ICD-10 diagnoses directly, this mapping represents a conservative approximation; individuals with qualifying self-reported disability who have never received a corresponding diagnosis would not be captured in claims under any algorithm design.

#### Channel B (claims visibility)

Among clinically eligible individuals, detection in claims data was modeled with race-specific probabilities parameterized from published evidence on differential healthcare utilization (White: 0.72; Black: 0.58; Hispanic: 0.61; AIAN: 0.52; Asian: 0.69).^7–9^ The AIAN parameter reflects well-documented fragmentation between the Indian Health Service and state Medicaid claims systems and lower ambulatory utilization among AIAN Medicaid enrollees.^15,16^ Nonmetropolitan enrollees received a detection penalty of 8% points based on evidence that rural Medicaid beneficiaries have 13%-18% lower inpatient care utilization than urban counterparts.^17^ States with HIE integration, ex parte determination, and shorter claims lag periods received detection probability bonuses (eAppendix B1) (To access the appendix, click on the Details tab of the article online).

#### Channel C (documentation burden)

In states requiring physician certification, successful documentation depended on certification probabilities that vary by race (White: 0.81; Black: 0.64; Hispanic: 0.67; AIAN: 0.55).^8,18^ Nonmetropolitan enrollees received a certification penalty of 6% points reflecting lower primary care provider density in rural areas. States with ex parte determination bypassed this channel.

For each state, racial/ethnic group, and metropolitan status, we ran 300 Monte Carlo replications with 2000 individuals sampled per group, yielding simulated frailty identification rates and 95% confidence intervals.

### Redesigned algorithm specification

The redesigned algorithm applied 4 evidence-based modifications, each with direct policy precedent in at least one existing state:

1. **Expanded diagnostic recognition**: ICD-10 families expanded to the California–New York union (13 families), adding social determinant codes Z59 (housing instability) and Z60 (social isolation). We acknowledge that Z-codes are differentially under-documented in administrative data—recent evidence demonstrates measurement bias in their recording across populations with varying levels of healthcare utilization, with Z-codes appearing less frequently in records of patients with lower baseline utilization even when social needs are present.^19^ We therefore present a sensitivity analysis excluding Z-codes from the expanded algorithm; results show the redesigned algorithm retains positive gains across all states without Z-codes.
2. **ADL threshold of 1** (federal 42 CFR 440.315 floor), lowered from the threshold of 2 used by Florida and Arizona. Lowering the ADL threshold expands the pool of individuals deemed clinically eligible under Channel A, increasing both the absolute count of identified individuals (numerator) and the pool eligible for detection. The denominator for all reported sensitivity rates is held constant as the full ACS-disabled adult population—individuals reporting at least one difficulty domain—under both status quo and redesigned algorithms, ensuring that apparent sensitivity gains reflect genuine identification improvement rather than denominator manipulation.
3. **Administrative data integration**: HIE, full ex parte determination, and claims lag under 3 months, modeled via a proportional gap closure framework in which these improvements close 40% of the gap between each population's baseline detection probability and near-perfect detection (0.98), based on evidence that HIE integration improves health record completeness and administrative data quality (eAppendix B2) (To access the appendix, click on the Details tab of the article online).^20,21^
4. **Elimination of physician certification**, consistent with current practice in Arkansas, Indiana, New York, California, Michigan, and Wisconsin.

### Head-to-head comparison

For each of 17 states, we ran the microsimulation under both the state's existing algorithm and the redesigned algorithm using identical ACS PUMS individual-level data. We compared overall sensitivity (percentage of ACS-disabled adults correctly identified), race-stratified sensitivity (Black-White, Hispanic-White, and AIAN-White gaps), and within-race rural-urban sensitivity differences. Sensitivity analysis varied detection and documentation probabilities by plus or minus 1 SD across 5 scenarios (eAppendix B3) (To access the appendix, click on the Details tab of the article online). Coverage impact projection assumptions (eAppendix B4), under-identification decomposition by channel (eAppendix B5), and the Z-code sensitivity analysis (eAppendix B6) are described in the appendix. Supplementary causal and algorithmic fairness analyses—staggered difference-in-differences,^22,23^ synthetic control case studies,^24^ and equalized-odds and calibration tests^25,26^—are presented in eAppendix C.

### Coverage impact projection

Additional frail adults identified under the redesigned algorithm were estimated as the sensitivity gain (percentage points) multiplied by each state's estimated expansion adult population. Coverage losses averted were estimated using the Arkansas 2018 benchmark disenrollment rate of 6.7%.^10^

All analyses used Python 3.10 with a fixed random seed of 42. Code is available at https://github.com/sanjaybasu/medicaid-frailty-bias.

### Limitations

Five limitations merit discussion. First, the ACS PUMS disability measure is self-reported and broader than state-specific ADL-based frailty criteria, meaning simulated sensitivity may overstate the under-identification gap relative to state-defined frailty. Second, the microsimulation relies on published estimates of race-differential detection and documentation probabilities; individual-level claims-linked disability data (available through ResDAC-restricted T-MSIS TAF files) would enable direct validation. Third, the proportional gap closure parameter (40%) is based on published evidence on HIE benefits.^20,21^ States with different health IT infrastructure may achieve different magnitudes of improvement. Fourth, the redesigned algorithm represents a composite of existing best practices, not a randomized intervention; projected gains assume that combining features from multiple states produces additive benefits. As partial external validation, we analyzed G2211 (“visit complexity inherent to E&M”) billing data from the same HHS dataset; among 8195 unique G2211-billing providers and 3.7 million claims, providers in specialties corresponding to the redesigned algorithm's 13 ICD-10 families (plus primary care) accounted for 52.6% of G2211 volume, with the highest specialist domains being genitourinary (3.9%), behavioral health (2.3%), cardiovascular (2.1%), and musculoskeletal (2.1%)—all included in the redesigned list (eAppendix D) (To access the appendix, click on the Details tab of the article online).^27^ Fifth, coverage loss projections apply the Arkansas 2018 disenrollment rate as a national benchmark, which may not generalize to states with different enforcement approaches.

## Results

### Study population

Across 17 study states, the estimated Medicaid expansion adult population aged 19-64 totaled approximately 30 million. The ACS PUMS sample included 75 043 Medicaid-enrolled adults with disability data across six domains. Mean ACS disability prevalence among Medicaid enrollees was 55.8%. State frailty policy stringency scores ranged from 2.4 (Florida, most restrictive) to 8.9 (California, most inclusive) (Table 1).

**Table 1:** State algorithm design features and sensitivity of Status quo vs redesigned frailty algorithms.

State	CER status	Stringency	ADL	Cert	HIE	SQ sensitivity (%)	Imp sensitivity (%)	Gain (pp)
Florida	Pending	2.4	2	Yes	No	14.3	40.7	+26.4
Arizona	Pending	2.8	2	Yes	No	14.3	40.7	+26.4
Tennessee	Pending	3.2	1	Yes	No	18.6	40.7	+22.2
Texas	Pending	3.5	1	Yes	No	23.5	40.7	+17.2
Arkansas	Terminated	3.8	1	No	No	30.5	47.2	+16.7
Oklahoma	Pending	4.1	1	Yes	No	24.6	44.0	+19.3
Georgia	Active	4.2	1	Yes	No	31.2	44.0	+12.8
Louisiana	Pending	4.8	1	No	No	29.0	44.0	+14.9
Kentucky	Blocked	5.0	1	Yes	No	31.2	44.0	+12.8
Ohio	Pending	5.3	1	No	No	36.7	44.0	+7.2
Indiana	Active	5.8	1	No	Yes	34.6	50.7	+16.1
Michigan	Blocked	5.9	1	No	No	32.9	48.8	+16.0
North Carolina	None	6.0	1	No	Yes	38.9	46.1	+7.2
Montana	Active	6.1	1	No	No	40.0	47.2	+7.2
Wisconsin	Blocked	6.4	1	No	Yes	43.7	50.7	+6.9
New York	None	8.4	1	No	Yes	45.4	51.8	+6.4
California	None	8.9	1	No	Yes	43.7	50.7	+6.9
**Mean (17 states)**						**31**.**4**	**45**.**6**	**+14**.**3**

Sensitivity = percentage of ACS-disabled Medicaid adults aged 19-64 correctly identified as medically frail via three-channel Monte Carlo microsimulation (300 replications, 2000 individuals per racial/ethnic group per state). States ordered by ascending policy stringency score. ADL = activities of daily living. Cert = physician certification required. HIE = health information exchange integration. SQ = status quo algorithm; Imp = redesigned algorithm. The redesigned algorithm applies: 13 ICD-10 diagnostic families (CA–NY union), ADL threshold = 1, HIE + full ex parte + short claims lag, no physician certification. CER = community engagement requirement. Bold values indicate the 17-state mean (overall summary row).

### Status quo algorithm performance

Existing state frailty algorithms identified a mean of 31.4% of ACS-disabled Medicaid adults as medically frail, ranging from 14.3% (Florida, Arizona) to 45.4% (New York) (Table 1). States with the most restrictive criteria—Florida (stringency 2.4), Arizona (2.8), and Tennessee (3.2)—identified fewer than 19% of disabled adults. States with more inclusive criteria—New York (8.4), California (8.9), and Wisconsin (6.4)—identified 43.7% or higher. Across all states, the majority of adults with ACS-defined functional disability were not identified by existing algorithms. Identification varied by race/ethnicity: mean White sensitivity was 39.1%, compared with 26.2% for Black, 28.9% for Hispanic, and 27.5% for AIAN enrollees (Table 1). Within race, metropolitan enrollees had 3%-5% point higher sensitivity than nonmetropolitan enrollees (eg, White metro: 41.4% vs White nonmetro: 36.7%).

#### Redesigned algorithm performance

**Figure 1:**
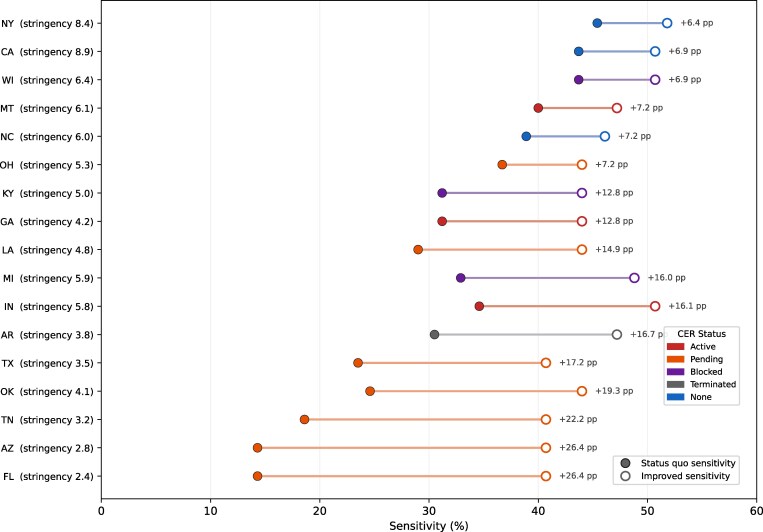
Overall sensitivity of status quo vs redesigned frailty algorithm, by state. Cleveland paired dot plot. Each state is represented by a status quo sensitivity estimate (filled circle) and redesigned algorithm sensitivity estimate (open circle), connected by a line indicating the gain in percentage points. States ordered by descending sensitivity gain. Color indicates CER status: red = active, orange = pending, purple = blocked, grey = terminated, blue = none. Estimated via three-channel Monte Carlo microsimulation on ACS PUMS 2022 data (N = 75 043 Medicaid adults; 300 replications, 2000 individuals per group per state). The redesigned algorithm applies: 13 ICD-10 diagnostic families, ADL threshold = 1, HIE + full ex parte, no physician certification.

The redesigned algorithm increased mean sensitivity to 45.6%, ranging from 40.7% (Florida, Arizona, and Tennessee) to 51.8% (New York) (Figure 1). All 17 states showed gains. The largest gains occurred in states with the most restrictive existing criteria: Florida and Arizona (+26.4% points each), Tennessee (+22.2), and Oklahoma (+19.3). States with already-inclusive criteria showed smaller but consistent gains: New York (+6.4) and Wisconsin and California (+6.9 each).

The improvement was driven primarily by the expanded diagnostic list and lowered ADL threshold (Channel A), which increased the pool of clinically eligible individuals from a mean of 49.8% to 55.8%. The proportional gap closure in detection probabilities (Channel B) and elimination of physician certification (Channel C) contributed additional gains. Sensitivity analyses varying detection and documentation parameters by plus or minus 1 SD confirmed that gains were positive under all scenarios (eAppendix B3) (To access the appendix, click on the Details tab of the article online).

### Equity evaluation

**Figure 2:**
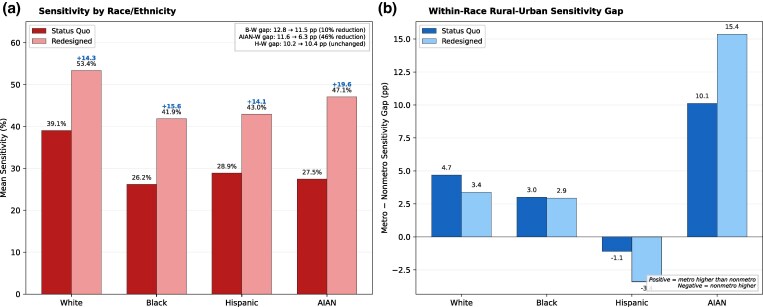
Multi-dimensional equity evaluation: sensitivity by race/ethnicity and rurality under status quo vs redesigned algorithm. (Panel a): Grouped bar chart of mean sensitivity (%) under status quo (dark) and redesigned (light) algorithms by racial/ethnic group (White, Black, Hispanic, and AIAN), showing absolute gains for each group. The AIAN-White gap narrowed from 11.6% to 6.3% points (46% reduction); the Black-White gap narrowed from 12.8% to 11.5% points (10% reduction); the Hispanic-White gap was essentially unchanged. (Panel b): Within-race rural-urban sensitivity gaps (percentage points, metro minus nonmetro) under status quo and redesigned algorithms. The rural penalty (3-5 pp for White and Black) persisted under both algorithms. The AIAN rural-urban gap widened under the redesigned algorithm, reflecting compounding of lower AIAN detection with the rural penalty.

The redesigned algorithm produced differential equity effects across racial/ethnic groups and geographies (Figure 2). The mean AIAN-White sensitivity gap narrowed from 11.6% to 6.3% points (46% reduction), the largest equity gain among subgroups evaluated. AIAN sensitivity increased by 19.6% points (from 27.5% to 47.1%), compared with 14.3 for White enrollees (from 39.1% to 53.4%). The mean Black-White gap narrowed modestly from 12.8% to 11.5% points (10% reduction), with Black sensitivity increasing by 15.6% points (from 26.2% to 41.9%). The Hispanic-White gap was essentially unchanged (10.2% to 10.4% points), as Hispanic enrollees gained 14.1% points, comparable to White gains.

Within race, the rural-urban sensitivity gap narrowed for White enrollees (from 4.7% to 3.4% points, metro higher) and was stable for Black enrollees (3.0% to 2.9% points). However, the AIAN rural-urban gap widened (from 10.1% to 15.4% points), reflecting the compounding of lower AIAN detection with the rural penalty; importantly, both AIAN metro (30.6% to 51.8%, +21.2 pp) and AIAN nonmetro (20.5% to 36.4%, +16.0 pp) enrollees showed substantial absolute gains, indicating that the redesigned algorithm improves identification for all AIAN subgroups even as the gap between them widens. The aggregate rural-urban gap was modest, as the disproportionately White composition of rural areas offset within-race rural penalties.

### Projected coverage impact

Adoption of the redesigned algorithm would identify an estimated 3 773 268 additional medically frail adults across 17 states, averting an estimated 252 799 coverage losses under full community engagement requirement enforcement (Table 2). The largest absolute impact was projected in Florida (667 666 additional identified; 44 733 losses averted), Texas (586 500; 39 295), and California (555 676; 37 230). Even states with relatively inclusive existing algorithms—such as New York and Wisconsin—would gain, reflecting the expanded diagnostic recognition and data integration improvements.

**Table 2:** Projected coverage impact of adopting the redesigned frailty algorithm, by state.

State	Expansion adults (est.)	Status Quo identified	Redesigned identified	Additional identified	Coverage losses averted
Florida	2 530 000	362 802	1 030 468	667 666	44 733
Texas	3 400 000	798 320	1 384 820	586 500	39 295
California	8 030 000	3 512 322	4 067 998	555 676	37 230
Arizona	1 210 000	173 514	492 832	319 318	21 394
New York	4 200 000	1 905 120	2 174 760	269 640	18 065
Georgia	1 403 000	437 455	616 758	179 303	12 013
Tennessee	780 000	144 690	317 693	173 003	11 591
Michigan	1 010 000	331 886	493 183	161 297	10 806
Louisiana	1 030 000	298 906	452 788	153 882	10 310
Indiana	902 000	311 821	456 953	145 132	9723
Oklahoma	620 000	152 830	272 552	119 722	8021
Kentucky	814 000	253 805	357 834	104 029	6969
North Carolina	1 400 000	544 739	645 820	101 081	6772
Ohio	1 200 000	440 759	527 520	86 761	5812
Arkansas	512 600	156 548	242 049	85 501	5728
Wisconsin	850 000	371 790	430 610	58 820	3940
Montana	82 000	32 783	38 720	5937	397
**Total**	**29 974 000**			**3 773 268**	**252 799**

Additional frail adults identified = sensitivity gain (pp) × state expansion population. Coverage losses averted = additional identified × 6.7% (Arkansas 2018 benchmark disenrollment rate; Sommers et al. 2019). States ordered by additional identified (descending). Coverage loss projection assumes full community engagement requirement enforcement; limitations are discussed in eAppendix B4 (To access the appendix, click on the Details tab of the article online). Bold values indicate the 17-state totals (Expansion adults, Additional identified, Coverage losses averted).

## Discussion

### Principal findings

Existing Medicaid frailty algorithms identify fewer than one-third of adults with functional disability as medically frail, with a mean sensitivity of 31.4% across 17 state algorithms. An evidence-based redesigned algorithm incorporating expanded diagnostic criteria, health information exchange integration, ex parte determination, and elimination of physician certification increased mean sensitivity to 45.6% (+14.3% points). The redesigned algorithm halved the AIAN-White identification gap and modestly narrowed the Black-White gap. Within-race rural-urban differences persisted, but in a context of increased sensitivity for all categories, such that there would be absolute improvements for all categories were the new algorithm to be implemented. These improvements would identify nearly 3.8 million additional frail adults and avert approximately 253 000 coverage losses under the One Big Beautiful Bill Act mandate.

### Mechanism

Three design features of existing algorithms contribute to under-identification. First, restrictive ICD-10 diagnostic lists miss conditions prevalent among working-age Medicaid enrollees—particularly musculoskeletal disorders, broadly-defined nervous system conditions, and social determinant codes that California and New York recognize but most states do not. Second, reliance on MMIS claims data without HIE supplementation systematically misses individuals whose care is fragmented across providers or occurs in settings that generate fewer qualifying codes—a mechanism with well-documented racial differentials.^7–9^ Third, physician certification requirements impose procedural barriers that compound access differentials across communities with varying primary care capacity.^8,18^

The heterogeneous equity effects reflect how algorithmic redesign interacts with different structural barriers. The large AIAN-White gap reduction (46%) occurs because AIAN populations, with the lowest baseline detection probability (0.52), had the most room for proportional improvement when data fragmentation between IHS and state Medicaid systems is addressed. The modest Black-White gap reduction (10%) reflects that while Black enrollees gained more in absolute terms (+15.6 vs +14.3% points for White), the expanded diagnostic criteria and lowered ADL threshold—which have similar impacts across racial categories—contributed the majority of gains, partially offsetting the race-differential detection improvement. The persistent within-race rural-urban gap suggests that data integration alone does not fully compensate for lower provider density in nonmetropolitan areas; targeted rural outreach or telehealth strategies may be needed alongside algorithmic reform.

This finding is consistent with evidence that HIE integration improves health record completeness and care quality: a systematic review found beneficial effects in 90% of HIE quality-of-care analyses,^20^ and Indiana's statewide HIE improved race data completeness in administrative records from 38% to 60%.^21^ Findings are also consistent with experimental evidence that Medicaid coverage produces measurable health effects.^28^ Prior work has focused on documenting racial unfairness in health algorithms.^7^ These results suggest that targeting overall algorithm performance yields the largest equity co-benefit for the most marginalized populations (AIAN, rural AIAN) but that race-neutral reforms alone are insufficient to fully close identification gaps driven by structural access disparities.

### Policy implications

Three implications follow. First, CMS retains authority under 42 C.F.R. § 430.25 and Public Law 119-21 rulemaking provisions to establish minimum algorithmic design standards for state frailty determination systems.^3^ The difference between the least and most inclusive existing algorithms represents a 30% point gap in identification sensitivity. Specific minimum standards should include the following: (1) recognition of at least 10 ICD-10 diagnostic condition families (the current range spans 5-13 across states); (2) mandatory health information exchange integration to supplement MMIS claims data; (3) full ex parte determination capability—automated screening without requiring individual application—to reduce procedural barriers; (4) claims adjudication lag under 3 months; and (5) elimination of physician certification requirements in favor of claims-based identification, consistent with current practice in Arkansas, Indiana, New York, California, Michigan, and Wisconsin. Second, HIE integration should be a minimum adequacy standard rather than an optional design feature: states with HIE integration achieved substantially higher sensitivity even before algorithmic redesign. Third, physician certification requirements should be eliminated in favor of data-driven ex parte determination, consistent with practice in 6 states, to remove a documentation barrier that disproportionately affects enrollees in communities with lower provider density. Supporting evidence from staggered difference-in-differences and synthetic control analyses of community engagement requirement implementation is presented in eAppendix C (To access the appendix, click on the Details tab of the article online).

## Conclusion

Existing Medicaid frailty algorithms miss the majority of functionally disabled adults, with identification gaps that disproportionately affect AIAN, Black, and rural enrollees. An evidence-based redesign incorporating expanded diagnostic criteria, HIE integration, ex parte determination, and elimination of physician certification improves identification across all populations and narrows racial disparities—particularly for AIAN enrollees—as a co-benefit, although within-race rural-urban gaps warrant complementary interventions, consistent with broader evidence that Medicaid coverage improves health outcomes.^29^ CMS should consider establishing minimum algorithmic design standards for state frailty determination systems under the One Big Beautiful Bill Act.

## Reporting guideline

STROBE (Strengthening the Reporting of Observational Studies in Epidemiology) and RECORD (REporting of studies Conducted using Observational Routinely-collected health Data) extension^13,14^

## Supplementary Material

qxag108_Supplementary_Data

## Data Availability

All analysis code is available at https://github.com/sanjaybasu/medicaid-frailty-bias. Primary data sources are identified in Methods. Program status data reflect administrative records available as of early 2024 and may have changed subsequently.
