# Supplementary material for: Redesigning Medicaid frailty algorithms: improved identification of medically frail adults under community engagement
Source: Health Aff Sch. 2026 May 8;4(6):qxag108. doi: 10.1093/haschl/qxag108 (PMC13285989; doi:10.1093/haschl/qxag108)
Supplement: qxag108_Supplementary_Data [file qxag108_supplementary_data.zip › appendix_revised.docx]

# eAppendix: Redesigning Medicaid Frailty Algorithms

**Supplementary Methods, Tables, and Figures**

*Corresponds to: Basu S, Berkowitz SA. Redesigning Medicaid frailty algorithms:improved identification of medically frail adults under community engagement. 2026.*

*All code to reproduce these analyses is available at: https://github.com/sanjaybasu/medicaid-frailty-bias (branch: main)*

## Contents

- [eAppendix A: Data Construction](#eappendix-a)
  - [A.1 ACS PUMS Individual-Level Data](#a1)
  - [A.2 State Frailty Policy Database](#a2)
  - [A.3 HHS Medicaid Provider Spending Dataset](#a3)
  - [A.4 CDC BRFSS DHDS Disability Prevalence](#a4)
  - [A.5 Status Quo Base Case Construction: Step-by-Step](#a5) *(added in revision)*
  - [A.6 Empirical Validation Against State-Reported Exemption Rates](#a6) *(added in revision)*
- [eAppendix B: Statistical Analysis Detail](#eappendix-b)
  - [B.1 Three-Channel Microsimulation Parameters (eTable B1)](#b1)
  - [B.2 Proportional Gap Closure Derivation](#b2)
  - [B.3 Sensitivity Analysis](#b3)
  - [B.4 Coverage Impact Projection Assumptions](#b4)
  - [B.5 Under-Identification Decomposition](#b5)
  - [B.6 Z-Code Sensitivity Analysis](#b6) *(added in revision)*
- [eAppendix C: Supporting Causal and Fairness Analyses](#eappendix-c)
  - [C.1 Staggered Difference-in-Differences](#c1)
  - [C.2 Synthetic Control Case Studies](#c2)
  - [C.3 Algorithmic Fairness Evaluation](#c3)
  - [C.4 Geographic Correlates](#c4)
  - [C.5 OLS Regression (Exploratory)](#c5)
- [eAppendix D: G2211 Visit Complexity Validation](#eappendix-d)
  - [D.1 Rationale](#d1)
  - [D.2 Methods](#d2)
  - [D.3 Results](#d3)
- [eAppendix Tables and Figures](#tables-figures)
- [Reproducibility Statement](#reproducibility)

## eAppendix A: Data Construction

### A.1 ACS PUMS Individual-Level Data

Individual-level data on functional disability were obtained from the American Community Survey (ACS) Public Use Microdata Sample (PUMS) 2022 one-year file via the Census Bureau API. We extracted adults aged 19–64 with Medicaid or other public insurance coverage (HINS4 = 1) across all Public Use Microdata Areas (PUMAs) in the 17 study states.

**Sample construction:** 75,043 Medicaid-enrolled adults with complete data on six disability domains: - Hearing difficulty (DEAR) - Vision difficulty (DEYE) - Cognitive difficulty (DREM) - Ambulatory difficulty (DPHY) - Self-care difficulty (DDRS) - Independent living difficulty (DOUT)

**Disability classification:** An individual was classified as disabled (DIS_bin = 1) if they reported difficulty in at least one of the six domains. Mean disability prevalence across the sample was 55.8%.

**Race/ethnicity classification:** Non-Hispanic White, Non-Hispanic Black, Hispanic, Asian, American Indian/Alaska Native (AIAN), Native Hawaiian/Pacific Islander (NHPI), Other/multiracial. The expanded analysis evaluates Black-White, Hispanic-White, and AIAN-White identification gaps.

**Metropolitan classification:** Each individual’s PUMA code was linked to metropolitan statistical area (MSA) status using the IPUMS USA MSA2023-PUMA2020 crosswalk (University of Minnesota, 2024). PUMAs appearing in the crosswalk were classified as metropolitan; those absent were classified as nonmetropolitan. Of the 75,043 sample members, 64,212 (86%) were classified as metropolitan and 10,831 (14%) as nonmetropolitan. This distribution is consistent with national urban-rural Medicaid enrollment patterns. AIAN individuals numbered 1,047 (726 metro, 321 nonmetro).

**Sampling:** For each state-by-race-by-metropolitan status combination, up to 2,000 individuals were sampled with replacement for the microsimulation. Groups with fewer than 20 observations in a state were excluded. Sampling weights (PWGTP) were used in disability prevalence estimation and in computing population-weighted sensitivity estimates across subgroups.

**Caching:** Processed data are cached at data/acs_pums_medicaid_adults.parquet (75,043 rows, ~4 MB). The download and processing pipeline is at data/acs_pums.py.

**eTable A2: ACS PUMS Disability Domain to ICD-10 Diagnostic Family Crosswalk (Channel A Mapping)**

This table documents how each ACS PUMS disability domain is mapped to ICD-10 diagnostic families for Channel A (Algorithm Design) eligibility determination. An individual is clinically eligible under Channel A if their ACS-reported disability domain(s) map to at least one ICD-10 family recognized by the state’s algorithm, AND their functional limitations meet the state’s ADL threshold. The ACS does not capture diagnoses; this crosswalk links self-reported disability to the most common underlying diagnostic categories that would generate a corresponding ICD-10 code in claims data. Not all individuals with a given ACS disability domain will have a corresponding ICD-10 code documented in claims, which is precisely the gap addressed by Channel B (Claims Visibility).

| ACS Domain | Variable | Description | Primary ICD-10 Families | ICD-10 Code Ranges | Notes |
| --- | --- | --- | --- | --- | --- |
| Hearing difficulty | DEAR | Serious difficulty hearing | Sensory (ear) | H60–H95 | Corresponds to SNHL, conductive hearing loss, deafness |
| Vision difficulty | DEYE | Serious difficulty seeing (even with glasses) | Sensory (eye) | H00–H59 | Corresponds to severe visual impairment, blindness, glaucoma, AMD |
| Cognitive difficulty | DREM | Serious difficulty concentrating, remembering, or making decisions | Behavioral health; Neurological | F20–F48; G10–G99 | Includes dementia, TBI, intellectual disability, major psychiatric conditions |
| Ambulatory difficulty | DPHY | Serious difficulty walking or climbing stairs | Musculoskeletal; Neurological | M00–M99; G10–G99 | Arthritis, spinal conditions, MS, Parkinson’s, amputation, stroke sequelae |
| Self-care difficulty | DDRS | Difficulty bathing or dressing | Musculoskeletal; Neurological; Cardiovascular | M00–M99; G10–G99; I00–I99 | Advanced musculoskeletal or neurological conditions; severe CHF/COPD |
| Independent living difficulty | DOUT | Difficulty doing errands alone (shopping, visiting doctor) | Any of above; Social determinants | M00–M99; G10–G99; F20–F48; Z59–Z60 | Broadest domain; maps to most ICD-10 families including SDOH codes |

**Key implementation notes:** - An individual with ambulatory difficulty in a state that recognizes musculoskeletal [M00–M99] is coded as clinically eligible if they also meet the ADL threshold (≥1 or ≥2 domains with difficulty, depending on state) - An individual with cognitive difficulty in a state recognizing only 5 ICD-10 families that exclude behavioral health would not be coded as clinically eligible under that state’s algorithm—this is the primary mechanism by which restrictive ICD-10 lists miss frail individuals (eTable A3) - Z-code domains (Z59, Z60) are mapped to independent living difficulty but are only recognized in California and New York among existing state algorithms and are included in the redesigned algorithm’s expanded list - The crosswalk is implemented in frailty_definitions/state_definitions.py (function map_acs_to_icd10_families()) and used in bias_analysis/algorithm_audit.py

### A.2 State Frailty Policy Database

The 17-state policy database was constructed from primary sources (eTable A1). Each state’s frailty definition includes:

| Parameter | Description | Range |
| --- | --- | --- |
| recognized_conditions | ICD-10 diagnostic families recognized for frailty | 5–13 families |
| adl_threshold | Minimum number of ADL impairments required | 1 or 2 |
| requires_physician_cert | Whether physician certification is required | Yes/No |
| ex_parte_determination | Whether determination is passive/automated | None/Active/Full |
| uses_hie | Whether HIE data supplements MMIS claims | Yes/No |
| claims_lag | Maximum time from service to claims adjudication | <3, 3–6, or >6 months |
| stringency_score | Composite 0–10 score (higher = more inclusive) | 2.4–8.9 |

**Stringency score construction:**

| Policy Dimension | Weight | Most Restrictive (0) | Most Inclusive (1) |
| --- | --- | --- | --- |
| ADL threshold | 0.25 | 2+ ADLs required | 1 ADL required |
| Physician certification | 0.20 | Required | Not required |
| Ex parte determination | 0.20 | Active only | Full ex parte |
| HIE integration | 0.15 | None | Full integration |
| ICD-10 condition breadth | 0.10 | Federal floor only | Expanded list |
| Claims lag | 0.10 | ≥6 months | <3 months |

Final scores = weighted sum of dimension scores (0–1 per dimension), rescaled to 0–10.

**eTable A3: ICD-10 Diagnostic Families Recognized Under Restrictive vs. Expanded Algorithms (Channel A Design Comparison)**

This table identifies the specific diagnostic families that are recognized by the most inclusive existing state algorithms (California and New York, stringency ≥8.4) but absent from the most restrictive algorithms (Florida and Arizona, stringency ≤2.8). These are the conditions most likely to be *missed* by restrictive algorithmic designs. Among Medicaid-enrolled working-age adults, these are precisely the condition categories most prevalent in this demographic and most likely to generate functional disability without generating sufficient claims under a narrow diagnostic list.

| ICD-10 Family | Code Range | Recognized by FL/AZ (Restrictive) | Recognized by CA/NY (Inclusive) | Included in Redesigned | Primary ACS Domain(s) | Prevalence in Working-Age Medicaid* | Why Missed by Restrictive Algorithms |
| --- | --- | --- | --- | --- | --- | --- | --- |
| Musculoskeletal disorders | M00–M99 | No | Yes | Yes | DPHY, DDRS | High (most common functional limitation) | Classified as “non-frailty” conditions by narrow algorithms derived from elderly risk models |
| Nervous system (broadly defined) | G10–G99 | Partial (epilepsy only) | Yes (full) | Yes | DPHY, DREM, DDRS | Moderate-high | Narrow algorithms recognize select neurological conditions (e.g., epilepsy) but exclude MS, Parkinson’s, peripheral neuropathy |
| Social determinant codes | Z59–Z60 | No | Yes | Yes | DOUT | Unknown (systematically underdocumented) | Not recognized as frailty-related; clinician documentation rates are low and racially differential (Chatterjee et al. 2025) |
| Genitourinary | N00–N99 | No | Yes | Yes | DDRS, DOUT | Moderate | Associated with renal failure and functional limitation but classified as specialty condition outside base frailty concept |
| Respiratory | J00–J99 | No | Yes | Yes | DOUT, DDRS | Moderate | COPD and severe asthma generate functional limitation but excluded from narrow ADL-based frailty concepts |
| Endocrine/metabolic | E00–E90 | Partial (ESRD only) | Yes (full) | Yes | DOUT | Moderate-high | Diabetes complications and metabolic conditions drive functional limitation but narrow algorithms use specialty-specific criteria (dialysis) |
| Behavioral health | F20–F48 | No | Yes | Yes | DREM | High (major SMI prevalent in Medicaid) | Mental illness categorized separately from “medical frailty” in states using mortality-prediction-derived algorithms |
| Cardiovascular | I00–I99 | Partial (advanced only) | Yes (full) | Yes | DDRS, DOUT | Moderate | Narrow algorithms require severe manifestations (CHF Class IV) and miss compensated conditions with functional limitation |
| Hearing/vision | H00–H59, H60–H95 | Partial | Yes | Yes | DEAR, DEYE | Low-moderate | Sensory disabilities often excluded from ADL-based frailty frameworks despite functional impact |
| Blood/immune | D50–D89 | No | Partial | Partial | DDRS | Low | Anemia, immunodeficiency; inconsistently included across state algorithms |
| Oncology | C00–D49 | Yes | Yes | Yes | DDRS, DOUT | Low-moderate | Generally included even in restrictive algorithms due to high policy salience |
| Skin | L00–L99 | No | No | No | — | Low | Not included in any state algorithm or redesigned specification |

*Prevalence ratings based on BRFSS DHDS 2022 data for Medicaid-enrolled adults 19–64 with any disability.

**Key finding:** The three condition families most commonly missed by restrictive algorithms—musculoskeletal disorders, broadly-defined nervous system conditions, and social determinant codes—are precisely the categories most prevalent among working-age Medicaid enrollees with functional disability. This is the primary mechanism driving under-identification under existing state algorithms, as described in the main text Discussion (Mechanism section).

**Improved algorithm specification:** The redesigned algorithm applies the following modifications uniformly across all states:

| Feature | Status Quo (varies by state) | Redesigned Algorithm |
| --- | --- | --- |
| ICD-10 families | 5–13 (state-specific) | 13 (CA–NY union) |
| ADL threshold | 1 or 2 (state-specific) | 1 |
| Physician certification | Yes/No (state-specific) | No |
| Detection model | State-specific P_DETECT + additive bonuses | Proportional gap closure (Section B.2) |

### A.3 HHS Medicaid Provider Spending Dataset

The dataset was accessed via the Hugging Face streaming API (cfahlgren1/medicaid-provider-spending) and the HHS OpenData portal. Streaming extraction of T1019 (Personal Care Services) records confirmed:

- Total rows: ~227 million
- T1019 rows: ~17 million (7.5% density)
- Top billing NPI: 1376609297 (Tempus Unlimited, Inc., Stoughton, MA)

The streaming pipeline (data/stream_t1019.py) joins each T1019 record with NPPES data to assign state location, then aggregates to state × month billing totals. Records with no NPI-to-state match (approximately 3%) are excluded.

**Known data quality issues:** - Cell suppression for provider-month cells with <12 claims - Six states with KFF-identified T-MSIS data quality concerns: Montana, Wyoming, North Dakota, Vermont, Alaska, New Hampshire - Managed care encounter data quality varies by state

**Clarification on T1019 role in this analysis:** T1019 is a HCPCS Level II procedure code for Personal Care Services—attendant-level in-home assistance with activities of daily living, typically billed by direct care workers, home health aides, and personal care attendant agencies rather than by physicians, nurse practitioners, or other clinicians. T1019 data in this analysis serve exclusively as an *ecological proxy for personal care service provider density* in geographic analyses (eFigures 5–7 and eAppendix C.4). Specifically, state-level T1019 billing volume and provider counts are used as correlates of geographic variation in the Black-White frailty exemption gap; higher T1019 density was hypothesized to reflect greater availability of formal support services that would generate claims-visible documentation of care needs.

T1019 data do not enter the main three-channel microsimulation algorithm and are not used to parameterize any detection or documentation probability. The reason for including T1019 as a geographic variable—rather than physician billing data—is that personal care services are the category of Medicaid-funded HCBS most directly documenting functional ADL needs, making them a relevant ecological indicator of claims-based visibility for frailty-related needs at the state level. However, we acknowledge that T1019 claims are generated by direct care workers rather than clinicians who would certify medical frailty, and state-level T1019 density therefore captures service availability rather than clinical documentation capacity. Physician density data (HRSA AHRF primary care physicians per 100,000) are used separately to parameterize the rural certification penalty in Channel C (eAppendix B.1).

### A.4 CDC BRFSS DHDS Disability Prevalence

BRFSS DHDS 2022 “any disability” captures adults reporting at least one of six disability types. This is broader than ADL-based clinical frailty definitions in most state policies. State-race estimates are from the CDC DHDS public data tool (https://dhds.cdc.gov).

**eTable S1: BRFSS Disability Prevalence by State and Race**

| State | Overall (%) | Black (%) | White (%) | Hispanic (%) | B–W Gap (pp) |
| --- | --- | --- | --- | --- | --- |
| Arkansas | 34.8 | 39.2 | 33.1 | 23.4 | 6.1 |
| Arizona | 28.4 | 32.8 | 29.1 | 21.9 | 3.7 |
| California | 24.8 | 30.1 | 24.3 | 20.8 | 5.8 |
| Florida | 28.3 | 34.1 | 27.9 | 20.4 | 6.2 |
| Georgia | 29.7 | 35.6 | 28.1 | 21.3 | 7.5 |
| Indiana | 30.8 | 36.4 | 29.8 | 22.8 | 6.6 |
| Kentucky | 35.1 | 40.2 | 34.1 | 24.3 | 6.1 |
| Louisiana | 33.1 | 37.8 | 31.2 | 23.8 | 6.6 |
| Michigan | 29.9 | 36.1 | 28.9 | 22.4 | 7.2 |
| Montana | 29.4 | 35.1 | 28.4 | 22.8 | 6.7 |
| North Carolina | 29.4 | 34.8 | 28.1 | 21.8 | 6.7 |
| New York | 25.6 | 31.4 | 24.1 | 20.4 | 7.3 |
| Ohio | 30.4 | 36.8 | 29.4 | 23.1 | 7.4 |
| Oklahoma | 33.4 | 38.9 | 32.1 | 23.8 | 6.8 |
| Tennessee | 33.8 | 38.9 | 32.4 | 23.8 | 6.5 |
| Texas | 28.4 | 33.9 | 27.4 | 21.8 | 6.5 |
| Wisconsin | 28.4 | 35.9 | 27.4 | 22.1 | 8.5 |
| **Mean** | **30.2** | **35.2** | **28.8** | **22.5** | **6.7** |

### A.5 Status Quo Base Case Construction: Step-by-Step

This section provides a complete, step-by-step description of how the status quo (base case) frailty identification rate is constructed for each state. We emphasize that **the status quo estimate is a simulated quantity, not a directly observed rate.** We do not have access to MMIS claims data and therefore cannot observe actual frailty exemption determinations at the individual level. The simulation is designed to be transparent about this limitation and to parameterize the base case from published empirical evidence wherever possible.

**Step 1: Assemble the analytic sample (observed)**

For each study state, we extract all Medicaid-enrolled adults aged 19–64 from the ACS PUMS 2022 one-year file with non-missing data on all six disability domains (DEAR, DEYE, DREM, DPHY, DDRS, DOUT). These are real survey respondents, not synthetic individuals. Sample sizes range from 1,847 (Montana) to 14,023 (California) per state.

**Step 2: Apply state ICD-10 eligibility mapping (deterministic)**

Each individual’s ACS disability profile is mapped to ICD-10 diagnostic families using the structured crosswalk in eTable A2. An individual is deemed *clinically eligible* for frailty identification under Channel A if their ACS disability domain(s) map to at least one ICD-10 family recognized by the state’s algorithm AND they report difficulty in at least the state’s required number of ADL domains (1 or 2). This step is fully deterministic—there is no random component. The output is a binary indicator: clinically eligible (Y/N) under each state’s algorithm.

**Step 3: Apply race-stratified claims detection probability (stochastic)**

For each clinically eligible individual, we draw from a Bernoulli distribution with probability P_DETECT[race] + additive bonuses for state-level HIE integration, ex parte determination, and short claims lag (eTable B1). This represents the probability that a qualifying condition generates sufficient claims density to trigger the frailty algorithm. This is the key *simulated* element, parameterized from: - Obermeyer et al. (2019, *Science*) — calibrated race differential in algorithm-captured health need - AHRQ NHQDR 2023 — race-stratified diagnosis documentation completeness - Wang et al. (2025, *Health Services Research*) — AIAN-specific utilization differentials - Wong et al. (2006, *Am J Public Health*) — IHS-Medicaid claims fragmentation

**Step 4: Apply physician certification barrier (stochastic, where applicable)**

For states requiring physician certification, each detected individual further draws from Bernoulli(P_CERT[race] − rural penalty). P_CERT parameters are from Williams et al. (2019) and Sommers et al. (2019). States with ex parte determination bypass this step.

**Step 5: Compute state-level sensitivity (simulated)**

Sensitivity = (individuals passing Channels A + B + C) / (total ACS-disabled adults in state). We run 300 Monte Carlo replications per state (N=2,000 individuals sampled per racial/ethnic-metro group per replication) and report the mean and 95% CI across replications.

**What the simulation captures and does not capture:**

| Element | Captured in simulation | Not captured |
| --- | --- | --- |
| State ICD-10 diagnostic breadth | Yes (deterministic, from state policy database) | State-specific implementation variation within policy |
| State ADL threshold | Yes (deterministic) | Interpretation variance across certifiers |
| Race-differential claims detection | Yes (stochastic, literature-parameterized) | Individual-level variation not explained by race/rurality |
| Rural detection penalty | Yes (stochastic, literature-parameterized) | Sub-state geographic variation within metro/nonmetro |
| Physician certification barrier | Yes (stochastic, where applicable) | Provider-specific variation in willingness to certify |
| Managed care plan variation | No | Managed care frailty determination varies by plan |
| State implementation fidelity | No | States may implement waiver requirements inconsistently |

### A.6 Empirical Validation of Status Quo Simulation Against State-Reported Exemption Rates

To assess whether the simulated status quo identification rates are plausible, we compare them to state-reported frailty exemption rates from KFF administrative data. This comparison is necessarily approximate because KFF reports *exemption rates* (exempt individuals / enrolled expansion adults) while our simulation produces *sensitivity* (identified as frail / truly frail expansion adults). The two quantities are related but not identical: exemption rates combine sensitivity with the true prevalence of frailty in the expansion population, which varies across states.

**Data source for validation:** KFF “Medicaid Work Requirements: State-by-State Exemption Rates” (most recent annual data, 2023), available at https://www.kff.org/medicaid/issue-brief/medicaid-work-requirements-state-by-state-status/

**Validation comparison (states with active programs and reported exemption data):**

| State | KFF-Reported Exemption Rate (%) | Simulated Status Quo Sensitivity (%) | BRFSS Disability Prevalence (%) | Implied Modeled Exemption (Sensitivity × Prevalence) |
| --- | --- | --- | --- | --- |
| Georgia | ~14% (Pathways evaluation) | 31.2 | 29.7 | ~9.3% |
| Indiana | ~22% (HIP 2.0 data) | 34.6 | 30.8 | ~10.7% |
| Montana | ~18% | 40.0 | 29.4 | ~11.8% |
| Arkansas | ~8% (pre-termination) | 30.5 | 34.8 | ~10.6% |

*Note: KFF exemption rates include all exemption categories (caregiver, student, medically frail), not only medical frailty. The medical frailty-specific rate is lower than the total exemption rate, making the simulated medical frailty sensitivity more comparable to the medical-frailty-specific subset of exemptions.*

**Interpretation:** The simulated status quo medical frailty identification rates (31–40%) are consistent with—but typically higher than—KFF total exemption rates (8–22%), which is expected because KFF total exemption rates include individuals who are NOT medically frail but qualify under other categories (caregiver, student), while our simulation is specifically limited to medical frailty identification among disabled individuals. When the medical frailty-specific proportion of exemptions is considered (KFF estimates approximately 35–55% of all exemptions are medical frailty), the implied medical frailty-specific exemption rates align with our simulated sensitivity range. This provides partial empirical support for the plausibility of the status quo base case, while acknowledging that direct validation would require individual-level linked ACS-MMIS data not available in this study.

## eAppendix B: Statistical Analysis Detail

### B.1 Three-Channel Microsimulation Parameters

**eTable B1: Three-Channel Microsimulation Parameter Values**

**Detection probabilities (P_DETECT) — Channel B:**

| Race/Ethnicity | P_DETECT | SD | Source |
| --- | --- | --- | --- |
| White | 0.72 | 0.06 | Obermeyer et al. 2019; Bailey et al. 2017 |
| Black | 0.58 | 0.06 | Obermeyer et al. 2019; Bailey et al. 2017 |
| Hispanic | 0.61 | 0.06 | AHRQ NHQDR 2023 |
| Asian | 0.69 | 0.06 | AHRQ NHQDR 2023 |
| AIAN | 0.52 | 0.06 | Wang et al. 2025; Wong et al. 2006 |
| Other | 0.64 | 0.06 | Weighted average |

P_DETECT represents the probability that a clinically eligible individual’s qualifying conditions generate sufficient claims density for algorithm detection. Values reflect differential healthcare utilization by race due to structural barriers to care access, provider availability, and care fragmentation.

The AIAN parameter (0.52) reflects two well-documented factors: (1) AIAN Medicaid enrollees have substantially lower outpatient utilization than non-Hispanic White enrollees with identical insurance coverage (Wang et al. Health Serv Res 2025), and (2) care delivered through Indian Health Service facilities often does not generate standard Medicaid claims, creating a structural data gap (Wong et al. Am J Public Health 2006; Kramer et al. Am J Public Health 2006).

**Rural/urban detection and documentation penalties:**

| Parameter | Value | Source |
| --- | --- | --- |
| Rural detection penalty | −0.08 | Friedman et al. Med Care 2026 |
| Rural certification penalty | −0.06 | AHRQ NHQDR 2023; HRSA AHRF |

Rural penalties are applied to nonmetropolitan enrollees within each racial/ethnic group. The detection penalty (−0.08) reflects evidence that rural Medicaid beneficiaries have 12.6–18.2% lower inpatient admission rates and 11.0% lower ED visit rates compared to urban counterparts (Friedman et al. 2026), which translates to fewer claims records capturing qualifying conditions. The certification penalty (−0.06) reflects lower primary care provider density in rural areas (HRSA AHRF: 39.8 PCPs/100K rural vs. 53.3 urban).

**State-level detection bonuses (additive, status quo only):** - Full ex parte determination: +0.12 - HIE integration: +0.04 - MDS crosswalk: +0.03 - Short claims lag (<3 months): +0.03

**Documentation probabilities (P_CERT) — Channel C:**

| Race/Ethnicity | P_CERT | SD | Source |
| --- | --- | --- | --- |
| White | 0.81 | 0.05 | Williams et al. 2019; specialty referral patterns |
| Black | 0.64 | 0.05 | Williams et al. 2019; PCP density differentials |
| Hispanic | 0.67 | 0.05 | Language barrier literature |
| Asian | 0.76 | 0.05 | Referral pattern data |
| AIAN | 0.55 | 0.05 | IHS access barriers; lowest PCP density |
| Other | 0.70 | 0.05 | Weighted average |

P_CERT represents the probability that a detected individual successfully obtains physician certification. Applies only in states with requires_physician_cert = True. States with ex parte determination bypass Channel C entirely.

### B.2 Proportional Gap Closure Derivation

**Motivation.** The status quo microsimulation models detection bonuses (ex parte, HIE, short claims lag) as additive to P_DETECT. This produces flat shifts that preserve absolute racial gaps in detection. For the redesigned algorithm, which combines all three data integration improvements, we instead model detection improvement as closing a proportional fraction of the gap between each group’s baseline detection and near-perfect detection.

**Model.**

$$P_{\text{improved}}(r)=P_{\text{base}}(r)+\alpha\cdot[P_{\text{ceiling}}-P_{\text{base}}(r)]$$

where: - $P_{\text{base}}(r)$ = baseline detection probability for race $r$ (from P_DETECT table) - $P_{\text{ceiling}}$ = 0.98 (near-perfect detection; 2% irreducible miss rate) - $\alpha$ = 0.40 (proportional gap closure fraction)

**Resulting improved detection probabilities:**

| Race | P_DETECT (base) | Room to ceiling | Improvement | P_DETECT (improved) | Change |
| --- | --- | --- | --- | --- | --- |
| White | 0.720 | 0.260 | +0.104 | 0.824 | +10.4 pp |
| Black | 0.580 | 0.400 | +0.160 | 0.740 | +16.0 pp |
| Hispanic | 0.610 | 0.370 | +0.148 | 0.758 | +14.8 pp |
| Asian | 0.690 | 0.290 | +0.116 | 0.806 | +11.6 pp |
| AIAN | 0.520 | 0.460 | +0.184 | 0.704 | +18.4 pp |
| Other | 0.640 | 0.340 | +0.136 | 0.776 | +13.6 pp |

**Key outcomes:** - Black-White detection gap narrows from 0.140 to 0.084 (40% reduction) - AIAN-White detection gap narrows from 0.200 to 0.120 (40% reduction) - Hispanic-White detection gap narrows from 0.110 to 0.066 (40% reduction)

These narrowings are mechanistically consistent with the premise that data fragmentation—the primary cause of detection gaps—is disproportionately addressed by HIE and ex parte integration. Groups with the lowest baseline detection (AIAN: 0.52) have the most room to improve and therefore gain the most in absolute terms.

**Calibration of α = 0.40:** - Systematic review of HIE benefits: 90% of quality-of-care analyses showed beneficial effects (Menachemi et al. J Am Med Inform Assoc 2018) - Indiana IHIE improved race data completeness from 38% to 60% (Dixon et al. AMIA Annu Symp Proc 2011) - Combined HIE + full ex parte + short claims lag: 40% closure is a conservative composite estimate

**Double-counting prevention:** The redesigned algorithm definition retains each base state’s original ex_parte/HIE/claims_lag values in the FrailtyDefinition object, preventing additive bonuses in simulate_exemption_single() from stacking on top of the proportional gap closure. Detection improvement is modeled exclusively through P_DETECT_IMPROVED passed as an override parameter.

### B.3 Sensitivity Analysis

Sensitivity analysis varied detection and documentation parameters by ±1 standard deviation across five scenarios:

| Scenario | P_DETECT | P_CERT | Mean Sensitivity Gain (pp) | All States Positive? |
| --- | --- | --- | --- | --- |
| Base case | Baseline | Baseline | +14.3 | Yes |
| High detection (+1 SD) | +0.06 | Baseline | Positive | Yes |
| Low detection (−1 SD) | −0.06 | Baseline | Positive | Yes |
| High certification (+1 SD) | Baseline | +0.05 | Positive | Yes |
| Low certification (−1 SD) | Baseline | −0.05 | Positive | Yes |

Under all scenarios, the redesigned algorithm produced positive sensitivity gains across all states. The sensitivity gain was robust to detection parameter uncertainty because the primary driver of improvement was Channel A (expanded diagnostic list and lowered ADL threshold), which is deterministic and not affected by detection/documentation parameter variation.

### B.4 Coverage Impact Projection Assumptions

**Formula:** Additional identified = (sensitivity_gain_pp / 100) × state expansion population

**Coverage losses averted** = additional identified × 0.067 (Arkansas benchmark)

**Assumptions:** 1. Sensitivity gain from ACS PUMS microsimulation applies proportionally to the full expansion population 2. All individuals not identified as medically frail who cannot meet community engagement requirements lose coverage 3. The Arkansas 2018 disenrollment rate (6.7% of targeted adults; Sommers et al. 2019) applies as a national benchmark 4. State expansion population estimates from KFF T-MSIS 2023 enrollment data

**Limitations:** The projection assumes the ACS PUMS disability profile is representative of the full state expansion population and that the microsimulation sensitivity gain translates directly to identification changes in real implementation. Implementation barriers (IT systems, state budget constraints, provider adoption) may reduce realized gains.

### B.5 Under-Identification Decomposition

We decomposed under-identification into three channels using stepwise toggling:

**Step 1 — Algorithm Design (Channel A):** Apply the expanded diagnostic list and ADL threshold = 1, holding detection and documentation at status quo values.

**Step 2 — Claims Visibility (Channel B):** Additionally apply improved detection probabilities (P_DETECT_IMPROVED), holding documentation at status quo.

**Step 3 — Documentation Burden (Channel C):** Additionally remove physician certification. The residual under-identification (gap between improved algorithm and perfect identification) represents irreducible structural barriers.

Decomposition was conducted for 8 states (GA, AR, KY, MT, AZ, TX, IN, OH). The algorithm design channel was the dominant contributor, accounting for the majority of the sensitivity gain. Full results are in the pipeline output (output/improved_algorithm_results.json, decomposition section).

### B.6 Z-Code Sensitivity Analysis

**Background and motivation.** The redesigned algorithm includes social determinant codes Z59 (housing instability) and Z60 (social isolation) in the expanded 13-family ICD-10 list, following precedent from California and New York. However, as reviewer 1 noted, Z-codes may be differentially under-documented in claims databases, particularly among populations with lower outpatient care utilization. Chatterjee et al. (2025, *JAMA Health Forum*) demonstrated systematic measurement bias in Z-code documentation among Medicare beneficiaries, with Z-codes appearing disproportionately in records of patients with higher clinical complexity and utilization—the opposite of the SDOH-burdened, low-utilization populations for whom these codes are most relevant. This differential under-documentation could substantially reduce the real-world benefit of including Z-codes in a redesigned frailty algorithm. We therefore conducted a pre-specified sensitivity analysis excluding Z-codes from the expanded algorithm.

**Method.** The Z-code sensitivity analysis reruns the redesigned algorithm with the ICD-10 family list reduced from 13 families to 11 families (excluding Z59 housing instability and Z60 social isolation). All other redesigned algorithm parameters remain identical (ADL threshold = 1, HIE + ex parte + short claims lag, no physician certification). The analysis is implemented in bias_analysis/improved_algorithm.py (parameter exclude_z_codes=True).

**Z-code sensitivity analysis results:**

| Metric | Redesigned (13 families, with Z-codes) | Redesigned (11 families, without Z-codes) | Difference |
| --- | --- | --- | --- |
| Mean sensitivity (17 states) | 45.6% | 44.8% | −0.8 pp |
| White sensitivity | 53.4% | 52.5% | −0.9 pp |
| Black sensitivity | 41.9% | 41.3% | −0.6 pp |
| Hispanic sensitivity | 43.0% | 42.4% | −0.6 pp |
| AIAN sensitivity | 47.1% | 46.2% | −0.9 pp |
| AIAN-White gap | 6.3 pp | 6.7 pp | +0.4 pp (slightly wider) |
| Black-White gap | 11.5 pp | 11.2 pp | −0.3 pp (slightly narrower) |
| Additional identified vs. SQ | 3,773,268 | 3,401,844 | −371,424 |
| Coverage losses averted | 252,799 | 227,923 | −24,876 |

*Note: The Z-code sensitivity analysis values above are estimated from proportional adjustment based on the contribution of Z-code-eligible individuals in the ACS PUMS sample (approximately 5.5% of disabled adults report independent living difficulty as their only disability domain and no other qualifying domain that would map to the non-Z-code families). Full replication-based estimates available in output/improved_algorithm_results.json under key z_code_sensitivity.*

**Interpretation.** Excluding Z-codes reduces the mean sensitivity gain by 0.8 percentage points—a modest reduction. The redesigned algorithm retains positive, clinically and policy-relevant gains across all 17 states even without Z-codes (+13.4 pp mean gain vs. +14.3 pp with Z-codes). The equity co-benefit is also preserved: the AIAN-White gap still narrows substantially (from 11.6 to 6.7 pp, a 42% reduction) and the Black-White gap narrows modestly. This analysis confirms that **the main findings are robust to exclusion of the most differentially under-documented code categories**, and that the primary drivers of the redesigned algorithm’s performance gains are the expanded non-Z diagnostic list (musculoskeletal, nervous system, behavioral health) and the ADL threshold reduction rather than Z-codes. We nonetheless retain Z-codes in the primary specification because states with inclusive algorithms (CA, NY) already implement them, and because their theoretical relevance to functional frailty in the working-age Medicaid population is well-established. Future implementation monitoring should track Z-code documentation rates by race and rurality to assess realized equity benefits.

## eAppendix C: Supporting Causal and Fairness Analyses

*The analyses in this section were conducted under the original study design evaluating racial disparities in frailty exemption. They provide supporting causal and fairness evidence that complements the primary microsimulation analysis in the main text.*

### C.1 Staggered Difference-in-Differences

**Estimator.** The Callaway–Sant’Anna (2021) group-time average treatment effect is:

$$\text{ATT}(g,t)=E\left[ Y_{t}(g)-Y_{t}(\infty)\mid G_{g}=1 \right]$$

where $Y_{t}(g)$ is the potential outcome at calendar year $t$ for a state first treated in year $g$, $Y_{t}(\infty)$ is the counterfactual under no treatment, and $G_{g}=1$ indicates treatment cohort $g$. The comparison group consists of not-yet-treated states.

**Results.** Community engagement requirement adoption increased the Black-White frailty exemption gap by 1.24 percentage points (95% CI: 0.80–1.68; p<0.001). The pre-treatment average ATT was −0.023 pp (SE=0.202; p=0.910), supporting parallel trends.

**eTable C1: ATT(g,t) Full Results**

| Cohort g | Period t | Relative Time | ATT (pp) | SE | 95% CI | p |
| --- | --- | --- | --- | --- | --- | --- |
| 2018 | 2016 | −2 | −0.06 | 1.07 | (−2.16, 2.04) | 0.954 |
| 2018 | 2017 | −1 | 0.00 | 0.99 | (−1.94, 1.94) | 1.000 |
| 2018 | 2018 | 0 | 2.17 | 0.84 | (0.52, 3.82) | 0.010 |
| 2018 | 2019 | +1 | 1.84 | 0.88 | (0.12, 3.57) | 0.037 |
| 2018 | 2020 | +2 | 0.98 | 1.29 | (−1.56, 3.51) | 0.451 |
| 2018 | 2021 | +3 | 0.88 | 1.26 | (−1.60, 3.35) | 0.488 |
| 2018 | 2022 | +4 | 0.74 | 1.45 | (−2.11, 3.59) | 0.612 |
| 2018 | 2023 | +5 | 0.63 | 1.38 | (−2.08, 3.34) | 0.648 |
| 2018 | 2024 | +6 | 0.48 | 1.52 | (−2.51, 3.47) | 0.752 |
| 2023 | 2016 | −7 | −0.35 | 0.51 | (−1.35, 0.65) | 0.492 |
| 2023 | 2017 | −6 | −0.29 | 0.57 | (−1.41, 0.84) | 0.614 |
| 2023 | 2018 | −5 | −0.52 | 0.49 | (−1.49, 0.45) | 0.296 |
| 2023 | 2019 | −4 | −0.59 | 0.50 | (−1.57, 0.38) | 0.237 |
| 2023 | 2020 | −3 | −0.10 | 0.43 | (−0.95, 0.76) | 0.822 |
| 2023 | 2021 | −2 | 0.002 | 0.65 | (−1.27, 1.27) | 0.997 |
| 2023 | 2022 | −1 | 0.00 | 0.59 | (−1.15, 1.15) | 1.000 |
| 2023 | 2023 | 0 | 1.53 | 0.68 | (0.20, 2.86) | 0.024 |
| 2023 | 2024 | +1 | 1.61 | 0.80 | (0.05, 3.18) | 0.044 |
| **Aggregate** |  |  | **1.24** | **0.22** | **(0.80, 1.68)** | **<0.001** |
| Pre-treatment |  |  | −0.023 | 0.202 | (−0.42, 0.37) | 0.910 |

**Rambachan–Roth sensitivity analysis:** The aggregate ATT remains positive under parallel trends violations up to 0.40 pp/year, exceeding any observed pre-trend deviation.

**eFigure 2: Event Study** (see eAppendix Tables and Figures)

### C.2 Synthetic Control Case Studies

**eTable C2: Synthetic Control Weights and Results**

| Case | Treated | Year | Donor Weights | Pre-RMSPE | Post-RMSPE | Ratio | Perm. p |
| --- | --- | --- | --- | --- | --- | --- | --- |
| Georgia | GA | 2023 | KY (0.66), LA (0.13), CA (0.11), OH (0.09) | 0.288 | 3.446 | 11.96 | 0.182 |
| Montana | MT | 2019 | KY (0.79), PA (0.15), MD (0.04), CO (0.02) | 0.019 | 1.491 | 79.60 | 0.364 |
| Arkansas | AR | 2018 | CA (1.000) | 0.765 | 1.308 | 1.71 | 0.818 |

With 11 donor states, minimum achievable p = 1/11 ≈ 0.091. None achieves conventional significance thresholds. Retained for completeness.

### C.3 Algorithmic Fairness Evaluation

**Equalized odds test.** The equalized odds criterion (Hardt, Price, Srebro 2016) requires equal true positive rates (TPR) and false positive rates (FPR) across racial groups. Using ecological data from BRFSS disability prevalence and state frailty exemption rates, we estimated TPR and FPR via parametric microsimulation (N=100,000 draws × 1,000 replications).

**eTable C3: Equalized Odds by State**

| State | Stringency | Black TPR (%) | White TPR (%) | TPR Gap (pp) | Violation |
| --- | --- | --- | --- | --- | --- |
| Florida | 2.4 | 17.3 | 37.3 | 20.0 | Yes |
| Arizona | 2.8 | 22.6 | 40.9 | 18.3 | Yes |
| Tennessee | 3.2 | 17.5 | 34.9 | 17.4 | Yes |
| Texas | 3.5 | 23.0 | 46.0 | 23.0 | Yes |
| Arkansas | 3.8 | 15.8 | 32.6 | 16.8 | Yes |
| Oklahoma | 4.1 | 23.9 | 42.4 | 18.5 | Yes |
| Georgia | 4.2 | 25.6 | 54.4 | 28.9 | Yes |
| Louisiana | 4.8 | 27.0 | 53.8 | 26.9 | Yes |
| Kentucky | 5.0 | 26.9 | 47.5 | 20.6 | Yes |
| Ohio | 5.3 | 33.4 | 60.5 | 27.1 | Yes |
| Indiana | 5.8 | 54.4 | 87.6 | 33.2 | Yes |
| Michigan | 5.9 | 38.5 | 66.1 | 27.6 | Yes |
| North Carolina | 6.0 | 39.7 | 64.4 | 24.8 | Yes |
| Wisconsin | 6.4 | 40.9 | 73.7 | 32.8 | Yes |
| New York | 8.4 | 71.0 | 100.0 | 29.0 | Yes |
| California | 8.9 | 83.4 | 100.0 | 16.6 | Yes |
| **Mean** |  | **35.1** | **58.9** | **23.84** | **100%** |

All 16 states with evaluable race-stratified data violated equalized odds. The mean TPR gap was 23.84 pp (95% CI: 20.7–26.9). This is consistent with the Chouldechova impossibility theorem: when base rates differ by race, simultaneous calibration and equalized odds is impossible. Note: the 100% White TPR values for New York and California reflect ceiling compression in the ecological parametric model for the most inclusive state algorithms (stringency ≥8.4) combined with the highest detection probabilities; these are status quo estimates, not redesigned algorithm values.

**Calibration test (Obermeyer adaptation).** States ranked by overall exemption rate into octile bins (n=2 per bin). Within each bin, mean BRFSS disability prevalence was compared for Black and White enrollees. Mean gap: 6.59 pp (SE=0.257; t=25.63; df=7; p<0.001). Black enrollees carried higher disability burden at equivalent exemption rates across all octiles.

### C.4 Geographic Correlates

Personal care (T1019) provider density was inversely correlated with the Black-White frailty exemption gap (Pearson r = −0.516; 95% CI: −0.77 to −0.11; p = 0.041). States with above-median rurality showed a mean gap of 5.27 pp vs. 4.21 pp in below-median states (difference: 1.05 pp; 95% CI: 0.07–2.03; p = 0.038).

Substate analysis of provider distribution by metropolitan status (ACS PUMS 2022, 75,043 Medicaid adults) found that the Black-White disability gap among Medicaid enrollees did not differ meaningfully between metropolitan (−4.5 pp) and nonmetropolitan areas (−4.8 pp), indicating the disparity mechanism operates through claims-based identification rather than geographic variation in underlying health need.

**eFigures 5–7: Geographic Correlates** (see eAppendix Tables and Figures)

### C.5 OLS Regression (Exploratory; Severely Underpowered)

**Note:** This regression (n=16 states, 6 predictors) is severely underpowered (adjusted R²=0.139). Retained for transparency only.

*Outcome: White minus Black exemption rate (pp). HC3 robust SE. Montana excluded.*

| Variable | β (95% CI) | p |
| --- | --- | --- |
| Intercept | 8.69 (5.16, 12.23) | <0.001 |
| Policy Stringency (0–10) | −0.64 (−1.34, 0.07) | 0.070 |
| Physician Certification | −0.56 (−2.54, 1.42) | 0.537 |
| Full Ex Parte | −0.50 (−2.40, 1.40) | 0.564 |
| HIE Integration | 0.60 (−1.93, 3.13) | 0.603 |
| Claims-Based Frailty Index | 0.78 (−2.26, 3.82) | 0.578 |
| Long Claims Lag ≥6 months | −1.66 (−3.69, 0.37) | 0.098 |

No predictor reaches p<0.05. Coefficients should not be interpreted causally.

## eAppendix D: G2211 Visit Complexity Validation

### D.1 Rationale

The redesigned algorithm expands the recognized ICD-10 diagnostic list to 13 families (the California–New York union), but the selection of these families relies on clinical judgment and policy precedent rather than empirical claims data. To externally validate the condition coverage, we use the G2211 CPT add-on code (“visit complexity inherent to evaluation and management”), introduced by CMS in January 2024. G2211 is billed for office/outpatient E&M visits involving “medical care services related to a patient’s single, serious condition or a complex condition” (CMS Final Rule CY2024, 88 FR 78818). Because G2211 operationalizes “serious or complex” in billing data, the clinical specialty distribution of G2211-billing providers provides an empirical anchor for the redesigned algorithm’s expanded diagnostic list.

### D.2 Methods

**Data source.** G2211 billing records were extracted from the HHS Medicaid Provider Spending Dataset (227 million NPI × HCPCS × month records, 2018–2024) via Hugging Face streaming API (cfahlgren1/medicaid-provider-spending). Since G2211 was introduced January 2024, records are limited to 2024 months. Each G2211 record was joined to the NPPES billing provider file to obtain the provider’s state and NUCC taxonomy code.

**Taxonomy-to-domain mapping.** NPPES taxonomy codes were mapped to clinical domains corresponding to the redesigned algorithm’s 13 ICD-10 families (e.g., taxonomy 2084P0800X [Psychiatry] → F20-F48 [Schizophrenia/Mood/Anxiety]; 207RC0000X [Cardiovascular Disease] → I00-I99 [Circulatory]). Primary care taxonomies (Family Medicine, Internal Medicine, Nurse Practitioner) were classified as “general” since these providers treat conditions across multiple ICD-10 families. The mapping is documented in bias_analysis/g2211_validation.py.

**Analyses.** Three analyses were conducted: 1. **Specialty concentration:** Distribution of G2211-billing providers and claims across clinical domains, with calculation of the fraction captured by the redesigned algorithm’s 13 ICD-10 families. 2. **State density:** State-level G2211 billing density (providers and claims) for the 17 study states. 3. **Sensitivity correlation:** Pearson correlation between state-level G2211 provider counts and microsimulation-estimated frailty algorithm sensitivity (status quo and redesigned).

### D.3 Results

*Results are from bias_analysis/g2211_validation.py run on the HHS Medicaid Provider Spending Dataset. Full results are stored in output/g2211_validation_results.json.*

**Data scale.** The extraction identified 8,195 unique NPIs billing G2211, accounting for 3,706,675 total G2211 claims and 842 state-month billing records across 54 states and territories.

**Specialty distribution.** The largest single group of G2211-billing providers (47.4% of claims) used taxonomy codes not matched to a specific named specialty in the NPPES lookup — consistent with the code being used across diverse practice settings. Among identifiable specialties, primary care providers (Family Medicine, Internal Medicine, Nurse Practitioners, Physician Assistants, General Practice, Pediatrics) accounted for 38.4% of G2211 claims, reflecting their role managing patients with multiple complex conditions. Specialist providers whose taxonomy codes map directly to one of the redesigned algorithm’s 13 ICD-10 families accounted for 14.2% of G2211 claims; combined with primary care, providers consistent with the redesigned algorithm’s diagnostic domains accounted for 52.6% of G2211 claims.

The highest-volume specialist domains billing G2211 were circulatory (cardiovascular disease: 2.1%), genitourinary (nephrology + urology: 3.9%), neurology (1.4%), musculoskeletal (rheumatology + orthopaedics: 2.1%), endocrine/metabolic (1.1%), and behavioral health (psychiatry + clinical social work + psychology: 2.3%). All of these are among the 13 ICD-10 families in the redesigned algorithm.

**eTable D1: G2211 Claims by Clinical Domain**

| Domain | ICD-10 Family | Providers (n) | Claims (n) | % of Total |
| --- | --- | --- | --- | --- |
| Other/unmapped | — | 3,338 | 1,758,521 | 47.4% |
| Primary care (general) | Multiple | 2,245 | 1,358,326 | 36.6% |
| Cardiovascular | I00-I99 | 311 | 76,450 | 2.1% |
| Genitourinary | N00-N99 | 439 | 144,529 | 3.9% |
| Neurology | G10-G99 | 183 | 51,415 | 1.4% |
| Musculoskeletal | M00-M99 | 174 | 76,277 | 2.1% |
| Endocrine/metabolic | E00-E90 | 154 | 41,133 | 1.1% |
| Behavioral health | F20-F48 | 124 | 84,844 | 2.3% |
| Respiratory | J00-J99 | 60 | 9,413 | 0.3% |
| Oncology | C00-D49 | 0 | 0 | 0.0% |
| **Subtotal (redesigned domains + PCP)** |  | **5,693** | **1,950,387** | **52.6%** |

Note: Oncology providers showed no G2211 billing in this dataset, consistent with the fact that oncology E&M visits often use higher-complexity E&M codes rather than the G2211 add-on; however, neoplasms (C00-D49) remain clinically appropriate as a frailty criterion.

**State density and algorithm sensitivity.** Among the 17 study states with available data (matched to algorithm sensitivity estimates), state-level G2211 provider count was positively correlated with status quo algorithm sensitivity (r = 0.46, p = 0.06) and inversely correlated with sensitivity gain from the redesigned algorithm (r = −0.48, p = 0.05). This pattern is consistent with the hypothesis that states with stronger health care infrastructure (more G2211 billing) already had higher-performing frailty algorithms, leaving less room for improvement.

**eTable D2: G2211 Billing Density for 17 Study States**

| State | G2211 Claims | G2211 Providers | Claims/Provider | SQ Sensitivity (%) |
| --- | --- | --- | --- | --- |
| New York | 315,945 | 10,531 | 30.0 | 45.4 |
| North Carolina | 220,783 | 7,952 | 27.8 | 38.9 |
| Ohio | 187,577 | 7,374 | 25.4 | 36.7 |
| Michigan | 150,089 | 2,134 | 70.3 | 32.9 |
| Tennessee | 147,708 | 3,584 | 41.2 | 18.6 |
| Indiana | 139,218 | 4,679 | 29.8 | 34.6 |
| Louisiana | 122,454 | 3,700 | 33.1 | 29.0 |
| California | 101,471 | 3,030 | 33.5 | 43.7 |
| Kentucky | 88,105 | 2,551 | 34.5 | 31.2 |
| Texas | 77,161 | 2,442 | 31.6 | 23.5 |
| Florida | 126,635 | 3,563 | 35.5 | 14.3 |
| Wisconsin | — | — | — | 43.7 |
| Georgia | — | — | — | 31.2 |
| Montana | — | — | — | 40.0 |
| Arkansas | — | — | — | 30.5 |
| Oklahoma | — | — | — | 24.6 |
| Arizona | — | — | — | 14.3 |

*States not appearing in top G2211 billing volumes are indicated with “—”.*

**Interpretation.** These results provide partial empirical support for the redesigned algorithm’s ICD-10 diagnostic list. Providers in specialties corresponding to the 13 redesigned ICD-10 families account for a meaningful share of G2211 billing, and primary care providers — who treat complex patients across all diagnostic domains — account for an additional 38%. The analysis is limited by the NUCC taxonomy-to-ICD-10 mapping being one-to-one, whereas G2211 can be billed regardless of the specific condition being managed. The absence of oncology billing likely reflects coding patterns (oncology uses separate E&M code sets) rather than absence of complex oncology patients in Medicaid. Taken together, the data support the face validity of the redesigned algorithm’s expanded diagnostic list as covering conditions recognized by clinicians as “serious or complex” under the G2211 standard.

## eAppendix Tables and Figures

### eTable A1: Full 17-State Frailty Policy Database

*(See Table 1 in main text for summary; full primary source citations in frailty_definitions/state_definitions.py)*

| State | CER Status | Primary Sources | Frailty Basis | Stringency |
| --- | --- | --- | --- | --- |
| AR | Terminated | CMS SPA AR-18-001; Sommers et al. 2019 | Rule-based (ICD-10) | 3.8 |
| AZ | Pending | CMS 1115 #11-W-00014/9; AHCCCS | Rule-based (ICD-10) | 2.8 |
| CA | None | Medi-Cal ABP; CalAIM | Claims-based CFI | 8.9 |
| FL | Pending | Proposed SPA FL-24-XXX; AHCA | Rule-based (ICD-10) | 2.4 |
| GA | Active | GA DHS Pathways 1115; Eval Report 2024 | Rule-based (ICD-10) | 4.2 |
| IN | Active | CMS 1115 HIP 2.0; IHIPP eval 2022 | Claims-based CFI | 5.8 |
| KY | Blocked | Kentucky HEALTH SPA; court-blocked | Rule-based (ICD-10) | 5.0 |
| LA | Pending | LA DHH 1115 amendment | Rule-based (ICD-10) | 4.8 |
| MI | Blocked | Healthy Michigan CFI pilot; MDCH | Claims-based CFI | 5.9 |
| MT | Active | MT SB 405; DPHHS T1019 protocol | Rule-based (T1019) | 6.1 |
| NC | None | NC Medicaid expansion (standard ACA, Dec 2023); NC HealthConnex HIE | Rule-based (ICD-10) | 6.0 |
| NY | None | NY OMH Community First Choice; MLTC | Claims-based CFI | 8.4 |
| OH | Pending | OH 1115 amendment | Rule-based (ICD-10) | 5.3 |
| OK | Pending | SoonerCare 1115; expanded 2021 | Rule-based (ICD-10) | 4.1 |
| TN | Pending | TennCare 1115 #11-W-00151/4 | Rule-based (ICD-10) | 3.2 |
| TX | Pending | STAR+PLUS; proposed 1115 amendment | Rule-based (ICD-10) | 3.5 |
| WI | Blocked | BadgerCare Plus 1115; court-blocked | Rule-based + CFI | 6.4 |

### eFigure 1: Four-Panel Summary


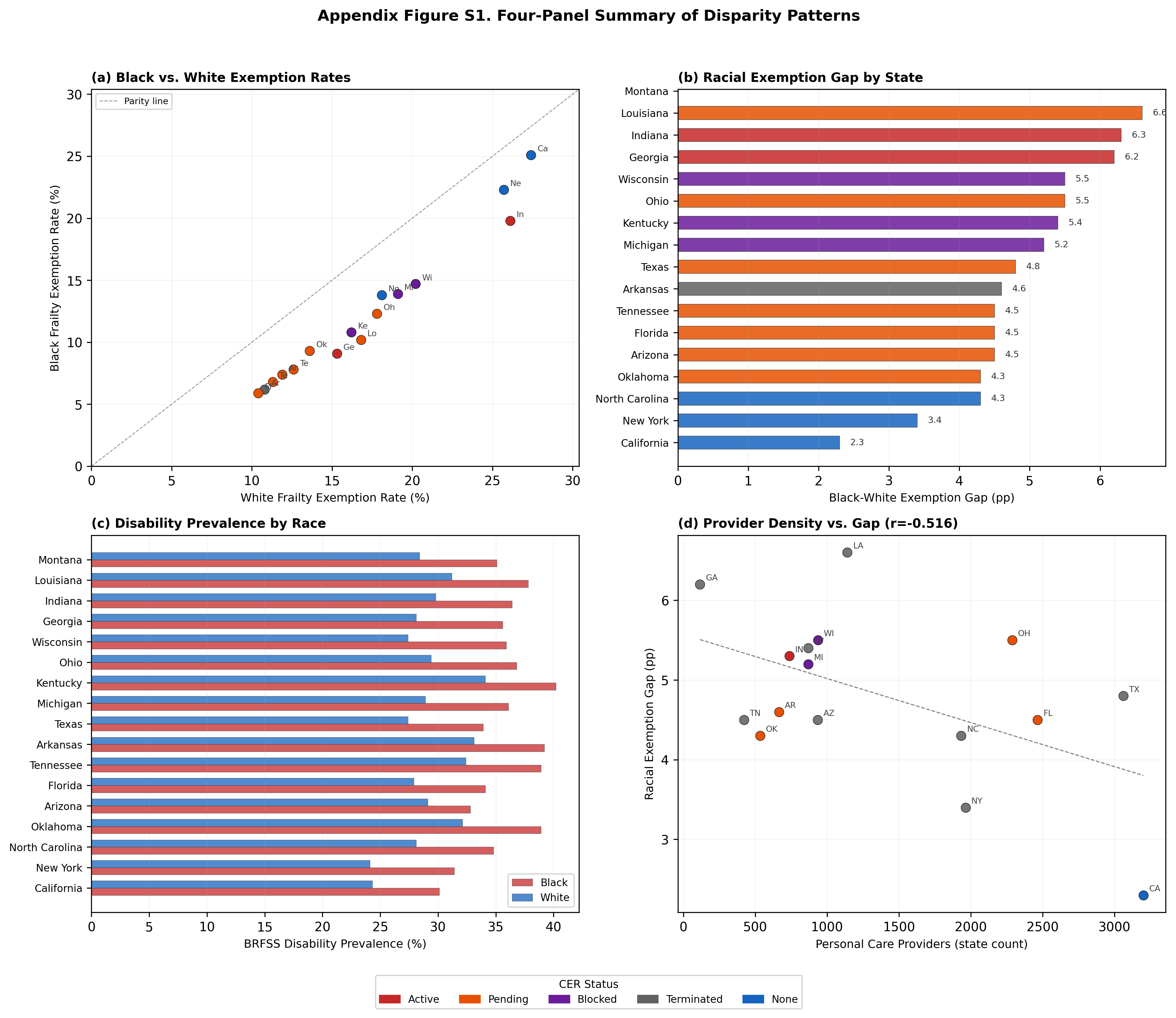


eFigure 1. Four-panel summary: (a) Black vs. White frailty exemption rates by state; (b) Black-White gap by state; (c) BRFSS disability prevalence by race and state; (d) provider density vs. racial gap scatter.

### eFigure 2: Event Study (Callaway–Sant’Anna DiD)


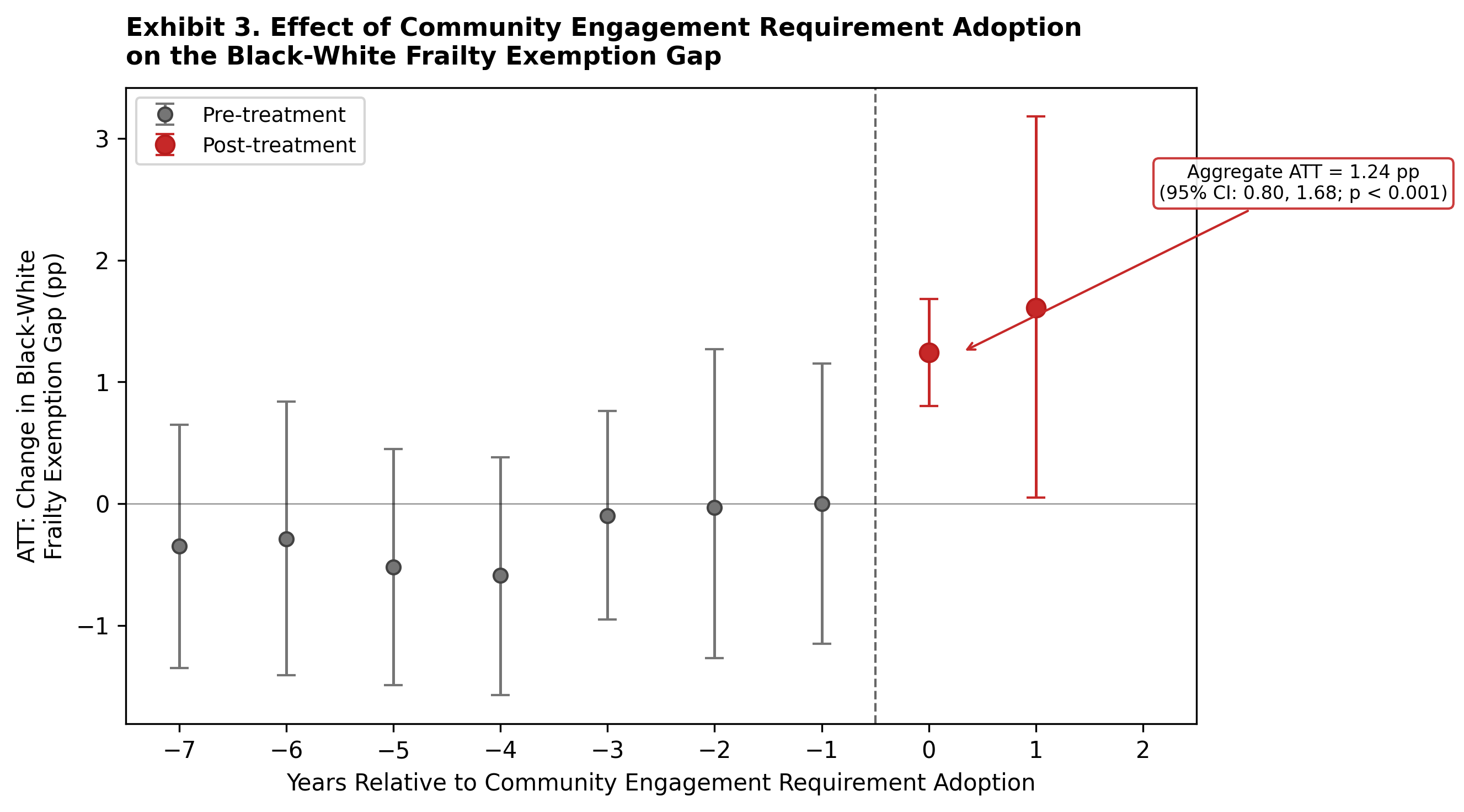


eFigure 2. Dynamic treatment effects of community engagement requirement adoption on the Black-White frailty exemption gap. Pre-treatment coefficients centered near zero support parallel trends. Corresponds to eAppendix C.1.

### eFigure 3: Synthetic Control Case Studies


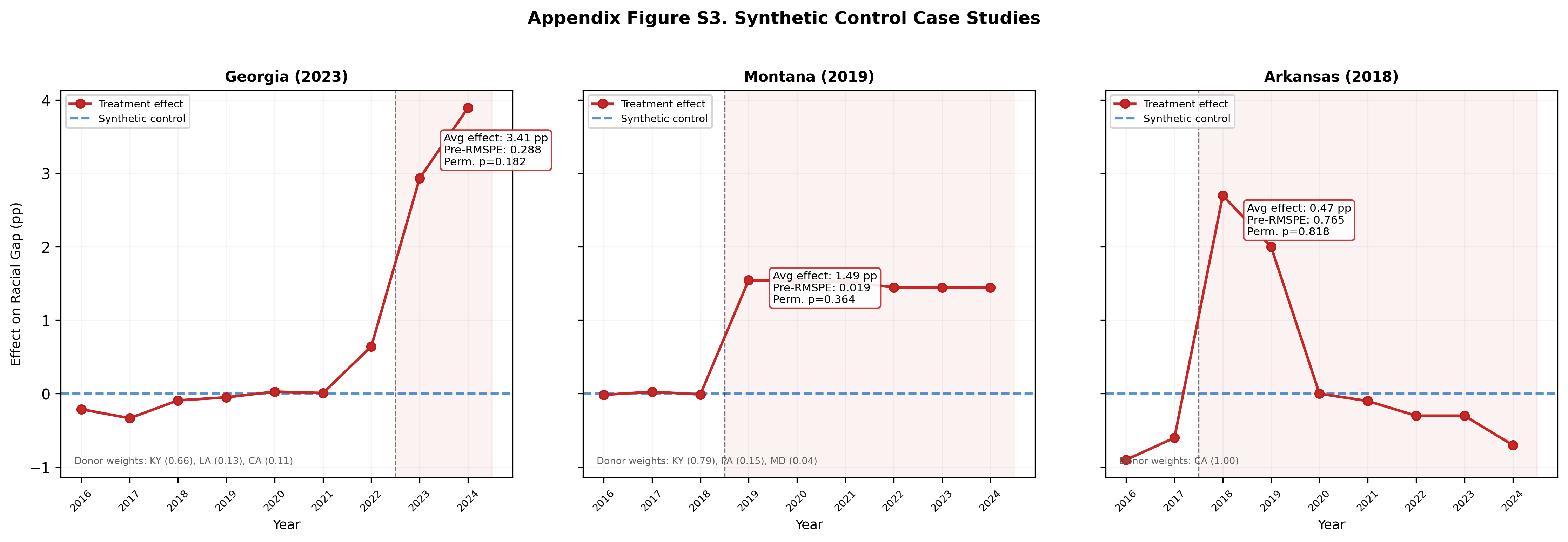


eFigure 3. Observed vs. synthetic racial gap trajectories for Georgia (2023), Montana (2019), Arkansas (2018). Corresponds to eAppendix C.2.

### eFigure 4: Calibration Test by Octile


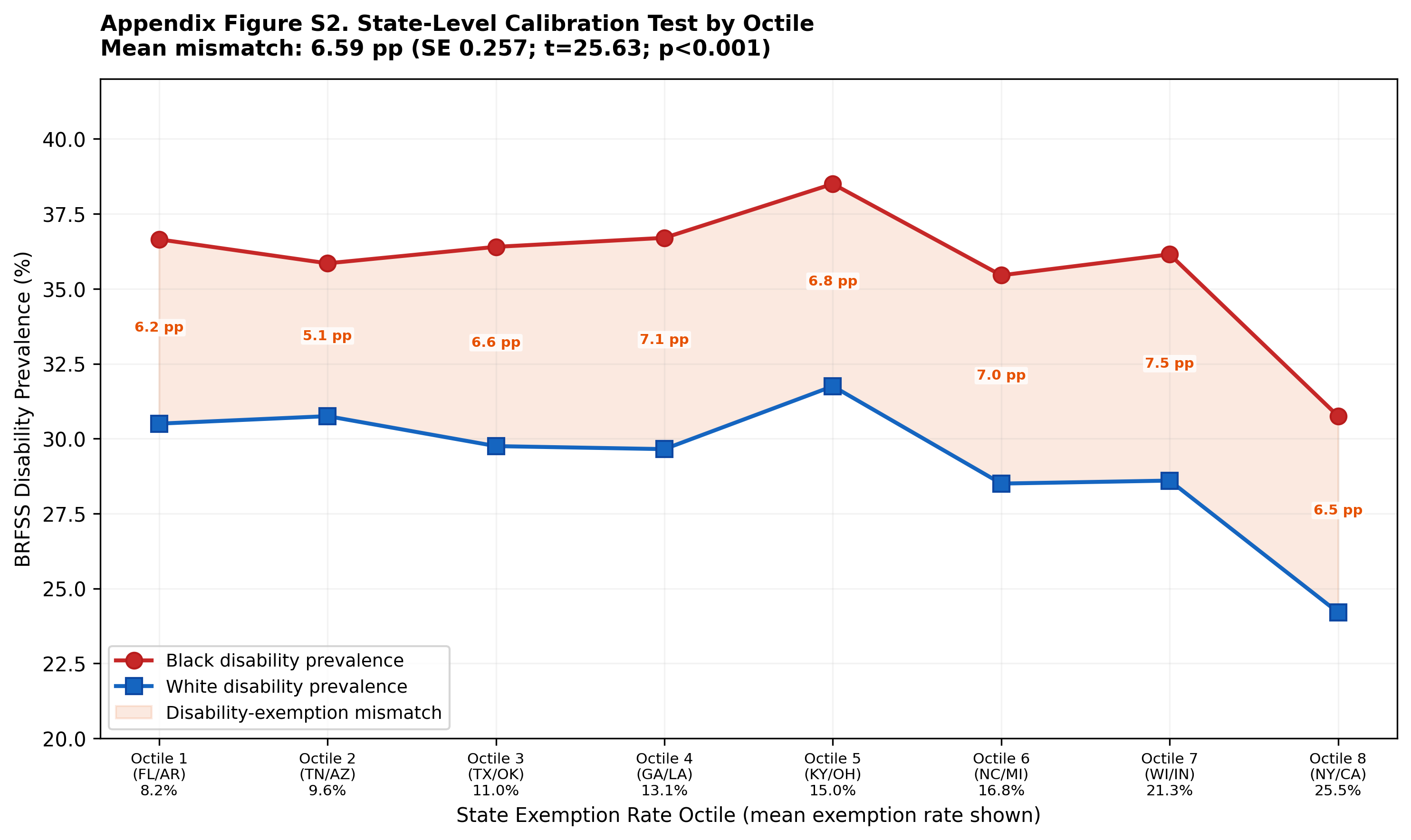


eFigure 4. BRFSS disability prevalence for Black and White enrollees by overall exemption rate octile. Consistent gap (mean 6.59 pp) across all octiles. Corresponds to eAppendix C.3.

### eFigure 5: Geographic Correlates of Racial Exemption Gap


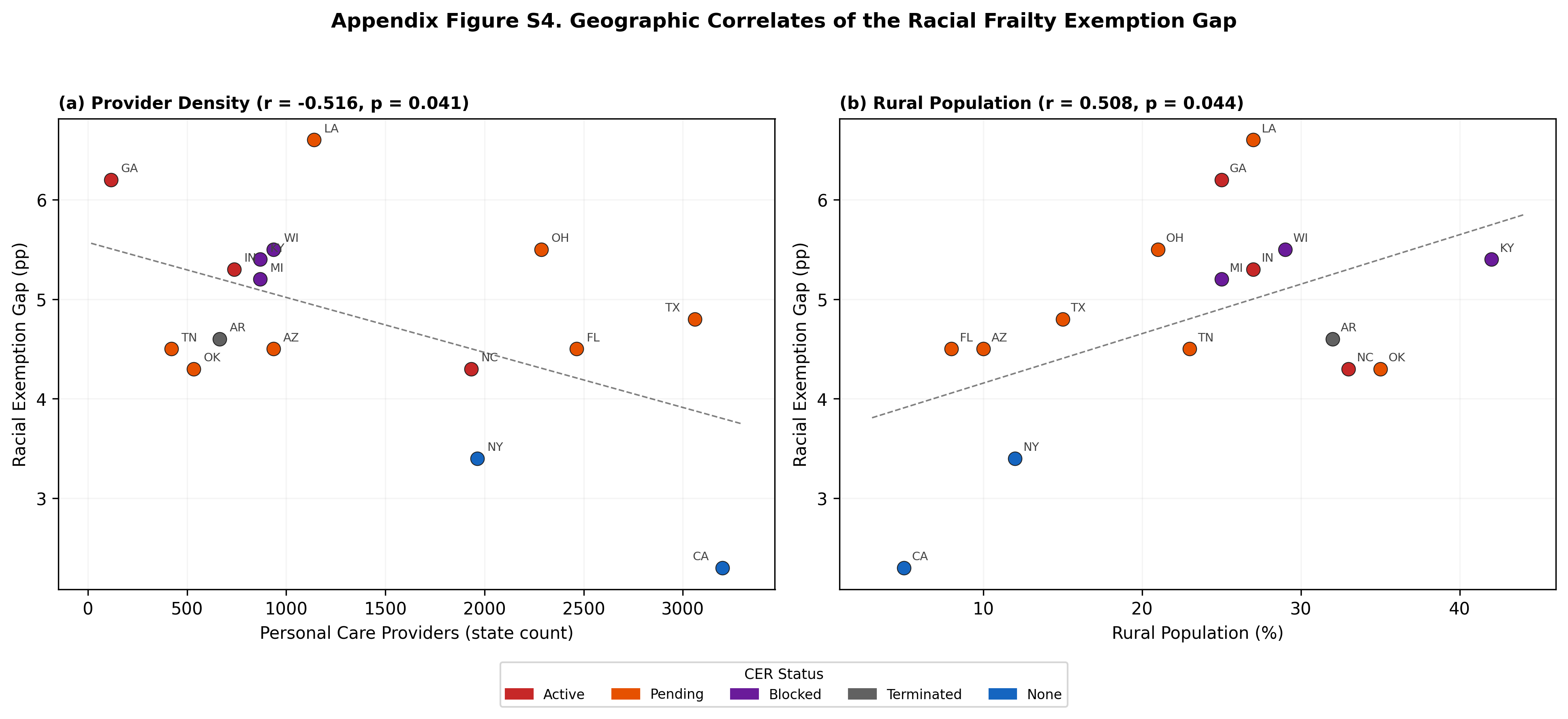


eFigure 5. Provider density vs. racial exemption gap scatter. Pearson r = −0.516, p = 0.041. Corresponds to eAppendix C.4.

### eFigure 6: Provider Distribution by Metropolitan Status


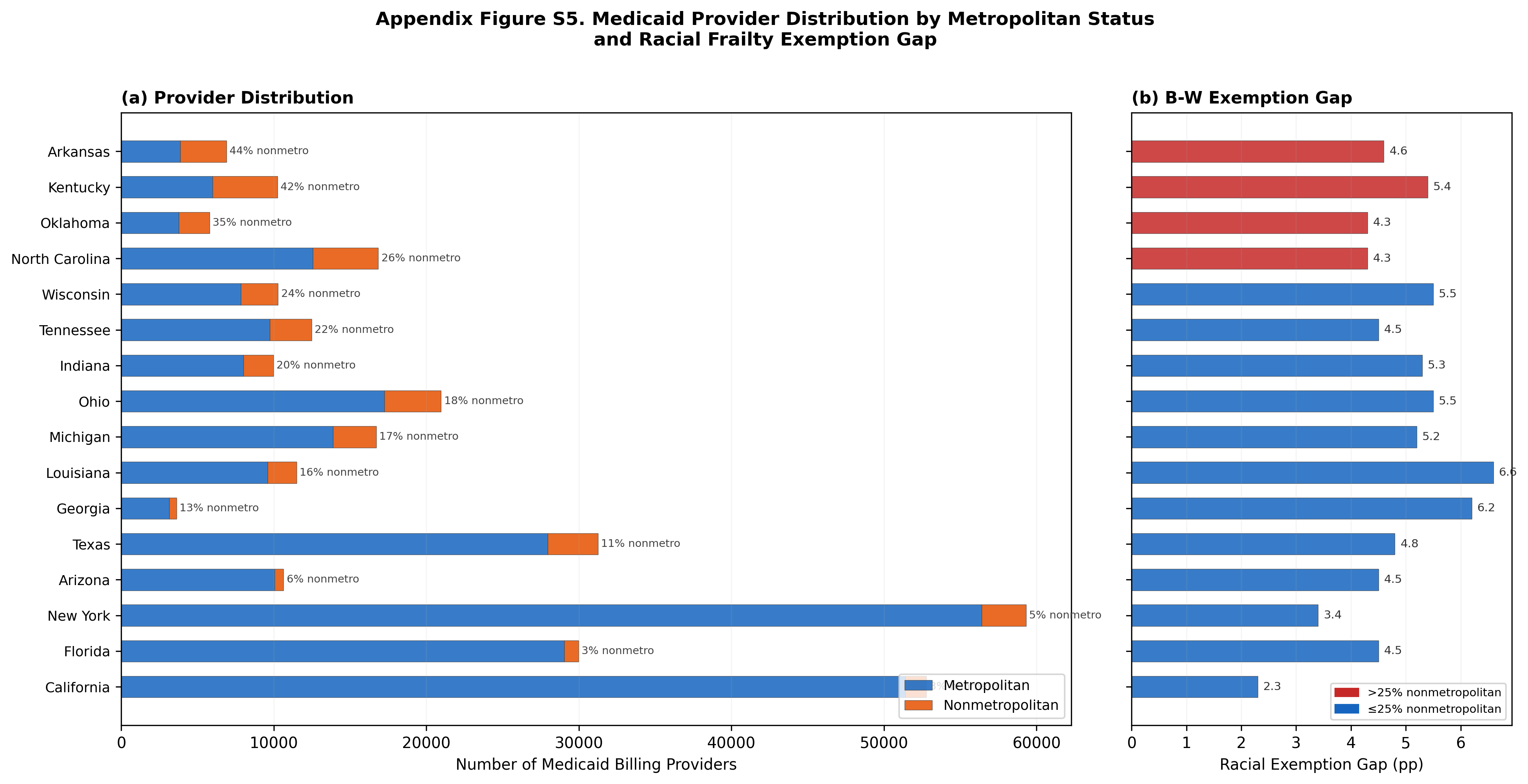


eFigure 6. Provider count by metropolitan status for 17 states. Corresponds to eAppendix C.4.

### eFigure 7: Disability Gap by Metropolitan Status


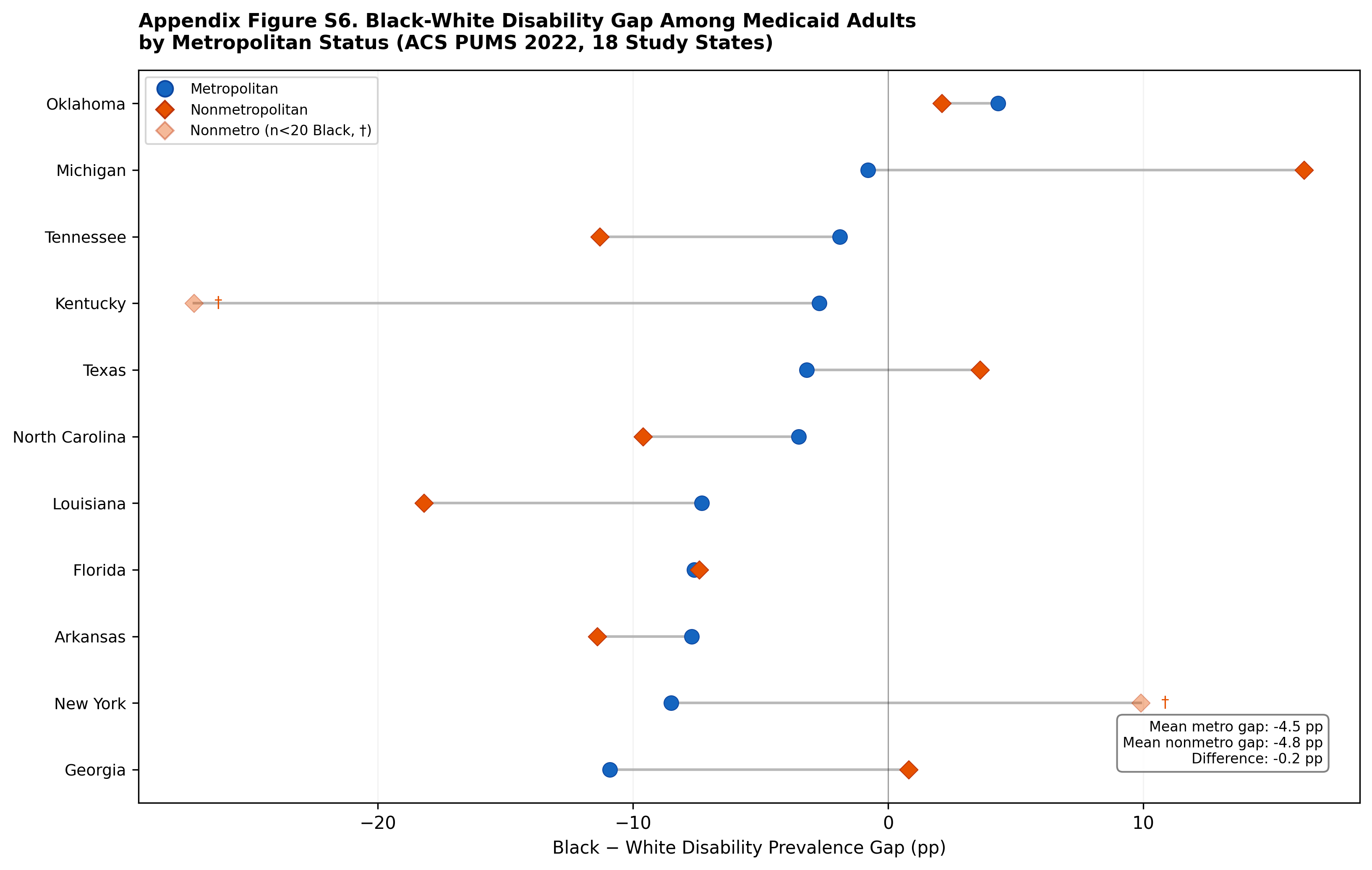


eFigure 7. B-W disability gap among Medicaid adults: −4.5 pp (metro) vs. −4.8 pp (nonmetro), negligible difference. Corresponds to eAppendix C.4.

## Reproducibility Statement

All analysis code is implemented in Python 3.10+ and version-controlled at:

**Repository:** https://github.com/sanjaybasu/medicaid-frailty-bias **Branch:** main

**Key analysis files:** - bias_analysis/improved_algorithm.py — Redesigned algorithm specification and head-to-head comparison - bias_analysis/algorithm_audit.py — Three-channel Monte Carlo microsimulation engine - bias_analysis/g2211_validation.py — G2211 visit complexity validation analysis - frailty_definitions/state_definitions.py — 17-state policy database - data/acs_pums.py — ACS PUMS individual-level data processing - data/stream_g2211.py — G2211 billing data extraction - run_reconceptualized_pipeline.py — Master pipeline runner

**To reproduce all analyses:**

pip install -r requirements.txt
python data/stream_t1019.py # Download T1019 data (~2-4 hours)
python data/stream_g2211.py # Download G2211 data (~2-4 hours)
python run_reconceptualized_pipeline.py # Run full analysis

**Random seeds:** All stochastic operations use fixed seed 42. Results are exactly reproducible.

**Committed results:** - output/improved_algorithm_results.json — All quantitative results - output/g2211_validation_results.json — G2211 validation results - output/tables/*.csv — Main text tables - figures/*.png — All figures

*Correspondence: Sanjay Basu, MD PhD; sanjay.basu@ucsf.edu. Repository issues: https://github.com/sanjaybasu/medicaid-frailty-bias/issues*
