# Supplementary material for: Redesigning Medicaid frailty algorithms: improved identification of medically frail adults under community engagement
Source: Health Aff Sch. 2026 May 8;4(6):qxag108. doi: 10.1093/haschl/qxag108 (PMC13285989; doi:10.1093/haschl/qxag108)
Supplement: qxag108_Supplementary_Data [file qxag108_supplementary_data.zip › icmje_disclosure.docx]

**ICMJE Form for Disclosure of Potential Conflicts of Interest**

(International Committee of Medical Journal Editors)

**Identifying Information**

| **Author name** | Sanjay Basu, MD PhD |
| --- | --- |
| **Manuscript title** | Redesigning Medicaid Frailty Algorithms: Improved Identification of Medically Frail Adults Under Community Engagement Requirements |
| **Journal** | Health Affairs Scholar |
| **Author role** | Co-first author; statistical analysis, study design, manuscript drafting |
| **Corresponding author?** | ☑ Yes ☐ No |

**Section 1: Work Under Consideration for Publication**

Did you or your institution at any time receive payment or services from a third party (government, commercial, private foundation, etc.) for any aspect of the submitted work (including but not limited to grants, data monitoring board, study design, manuscript preparation, statistical analysis, etc.)?

| **Grants** | ☐ Yes ☑ No (No external funding supported this study.) |
| --- | --- |
| **Personal fees** | ☐ Yes ☑ No |
| **Non-financial support** | ☐ Yes ☑ No |
| **Other** | ☐ Yes ☑ No |

**Section 2: Relevant Financial Activities Outside the Submitted Work**

In the past 36 months, have you received any of the following from entities that may have an interest in the general area of the submitted work? List all such relationships, including those of your spouse/partner. If none, check No for each item.

| **Grants** | ☑ Yes ☐ No • National Institutes of Health (research grants, outside the submitted work) • Centers for Disease Control and Prevention (research grants, outside the submitted work) |
| --- | --- |
| **Personal fees** | ☐ Yes ☑ No |
| **Non-financial support** | ☐ Yes ☑ No |
| **Other — employment/salary** | ☑ Yes ☐ No • Salary support from HealthRight360 (outside the submitted work) • Salary support from Waymark (outside the submitted work) |

**Section 3: Other Relationships or Activities**

Are there other relationships or activities that readers could perceive to influence, or that give the appearance of potentially influencing, what you wrote in the submitted work?

| **Other relationships** | ☑ Yes ☐ No • Board member and staff member at Waymark, a public benefit organization that provides free health and social services to patients receiving Medicaid. Neither author has a financial interest in the policy outcomes discussed in this manuscript. |
| --- | --- |

| **Signature:** | **Date:** |
| --- | --- |
| _______________________________________ | ____________________________ |

**ICMJE Form for Disclosure of Potential Conflicts of Interest**

(International Committee of Medical Journal Editors)

**Identifying Information**

| **Author name** | Seth A. Berkowitz, MD MPH |
| --- | --- |
| **Manuscript title** | Redesigning Medicaid Frailty Algorithms: Improved Identification of Medically Frail Adults Under Community Engagement Requirements |
| **Journal** | Health Affairs Scholar |
| **Author role** | Co-first author; study design, manuscript drafting and revision |
| **Corresponding author?** | ☐ Yes ☑ No |

**Section 1: Work Under Consideration for Publication**

Did you or your institution at any time receive payment or services from a third party (government, commercial, private foundation, etc.) for any aspect of the submitted work (including but not limited to grants, data monitoring board, study design, manuscript preparation, statistical analysis, etc.)?

| **Grants** | ☐ Yes ☑ No (No external funding supported this study.) |
| --- | --- |
| **Personal fees** | ☐ Yes ☑ No |
| **Non-financial support** | ☐ Yes ☑ No |
| **Other** | ☐ Yes ☑ No |

**Section 2: Relevant Financial Activities Outside the Submitted Work**

In the past 36 months, have you received any of the following from entities that may have an interest in the general area of the submitted work? List all such relationships, including those of your spouse/partner. If none, check No for each item.

| **Grants** | ☑ Yes ☐ No • National Institutes of Health (research grants, outside the submitted work) • North Carolina Department of Health and Human Services (research grant, outside the submitted work) • American Heart Association (research grant, outside the submitted work) • American Diabetes Association (research grant, outside the submitted work) |
| --- | --- |
| **Personal fees** | ☑ Yes ☐ No • Rockefeller Foundation (personal fees, outside the submitted work) • Johns Hopkins University Press (personal fees, outside the submitted work) |
| **Non-financial support** | ☐ Yes ☑ No |
| **Other** | ☐ Yes ☑ No |

**Section 3: Other Relationships or Activities**

Are there other relationships or activities that readers could perceive to influence, or that give the appearance of potentially influencing, what you wrote in the submitted work?

| **Other relationships** | ☐ Yes ☑ No Neither author has a financial interest in the policy outcomes discussed in this manuscript. |
| --- | --- |

| **Signature:** | **Date:** |
| --- | --- |
| _______________________________________ | ____________________________ |
